# Effects of constant and incremental modes of music volume and odor concentration on vigilance

**DOI:** 10.3389/fpsyg.2026.1810615

**Published:** 2026-04-07

**Authors:** Xinpo Ma, Jiaheng He, Haomiao Yu, Chenfei Wang, Rui Wang

**Affiliations:** School of Art, Hebei University of Science and Technology, Shijiazhuang, China

**Keywords:** dynamic incremental mode, HF-HRV, music volume, odor concentration, Psychomotor Vigilance Task, vigilance

## Abstract

**Introduction:**

Vigilance is critical in safety-critical domains, yet sustained attention often declines over time due to sensory adaptation. This study aimed to investigate whether dynamic incremental adjustments of music volume and odor concentration could counteract vigilance decrement more effectively than constant stimulation.

**Methods:**

A within-subjects design was adopted, in which 24 participants completed the Psychomotor Vigilance Task (PVT) under five experimental conditions. Subjective vigilance ratings (Karolinska Sleepiness Scale, KSS), behavioral performance indicators (reaction time and number of lapses), and physiological indicators (high-frequency heart rate variability, HF-HRV) were collected simultaneously.

**Results:**

Compared to the no-stimulus control condition, any condition involving music or odor was associated with significantly enhanced vigilance. For both auditory and olfactory modalities, the dynamic incremental mode was significantly superior to the constant mode in improving subjective alertness (lower KSS scores), shortening reaction time, reducing lapse count, and decreasing HF-HRV (indicating enhanced physiological arousal). When both modalities were presented incrementally, the optimal vigilance maintenance effect was observed, with most metrics showing significant improvements over the dual-constant condition and numerical trends consistently favoring the dual-incremental condition across all measures.

**Discussion:**

The core mechanism underlying this advantage is that the dynamic incremental mode, by increasing stimulus intensity in stages, effectively breaks the cycle of sensory adaptation induced by constant stimulation while dynamically maintaining arousal levels within the optimal range. The additive benefit observed under cross-modal dynamic input suggests enhanced regulation at the level of the autonomic nervous system. This study provides empirical evidence supporting the mechanisms by which dynamic multisensory stimulation counteracts vigilance decrement and offers theoretical support for designing adaptive, multimodal vigilance maintenance strategies in safety-critical domains such as driving and monitoring.

## 1 Introduction

Vigilance refers to the ability to sustain attention over extended periods and maintain a continuous state of alertness to specific external stimuli (Oken et al., 2006; Warm et al., 2008). It plays a critical role in key fields such as transportation, industrial safety, healthcare, work efficiency, and human factors engineering. Adequate vigilance not only enhances work performance but also safeguards personal safety (Caldwell et al., 2008). Consequently, in multiple domains where safety and efficiency are paramount, identifying effective methods to maintain or enhance individual vigilance has become an important research focus. According to the classic Yerkes–Dodson law, an individual's performance follows an inverted-U-shaped relationship with arousal level: both insufficient arousal and excessive arousal can impair vigilance and task execution, whereas a moderate level of arousal supports optimal cognitive functioning (Yerkes and Dodson, 1908; Rueda et al., 2023). Therefore, effective vigilance interventions should aim to dynamically regulate arousal toward this optimal zone. Currently, common vigilance-enhancing interventions include caffeine intake (Åkerstedt and Landström, 1998; Li et al., 2024), gum chewing (Allen et al., 2014; Schultheiss, 2013), and light exposure (Hsieh et al., 2022). However, these approaches suffer from limitations such as unstable effects, dependency issues, and restricted applicability in certain scenarios (Al-Shargie et al., 2019; Ru et al., 2019). In contrast, auditory and olfactory stimuli have gained increasing attention in recent years due to their advantages of being non-contact (Lahijanian et al., 2022), easy to implement, and having minimal side effects (Passos et al., 2022).

Music plays a significant role in the self-regulation of vigilance and cognitive abilities (Rickard et al., 2005; Chee et al., 2024), contributing to improved attention and task performance (Huang and Shih, 2011). However, music is a multidimensional stimulus: beyond acoustic features such as tempo and tonality, its semantic content (e.g., lyrics) and cultural familiarity can independently modulate listeners' cognitive and emotional states (Sun et al., 2024). Among these parameters, volume has been identified as a particularly critical factor influencing alertness, as it can be systematically manipulated without altering the musical content itself. From a neurophysiological perspective, the alerting effect of auditory stimuli is closely linked to the locus coeruleus-norepinephrine (LC-NE) system, which plays a central role in modulating arousal and attentional gain (Sara, 2009). Research by Scott et al. (2025) demonstrates that appropriate music can help sustain vigilance during repetitive or sustained-attention tasks. Among various acoustic parameters, volume is a key factor influencing alertness. Bishop et al. (2009) found that participants listening to higher-volume music showed significantly faster reaction times in choice reaction tasks and reported higher subjective alertness compared to those exposed to moderate volume levels. However, excessively high volume can also impair vigilance. Dalton et al. (2008) revealed that overly loud music leads to slower reaction and movement times, as well as reduced driving performance. At low volume levels, the influence of music genre becomes more complex; for instance, hard rock may improve reaction times in some tasks but could increase collision risk in simulated driving. Further supporting this, Turner et al. (1996) conducted reaction-time experiments and concluded that moderate volume effectively maintains auditory vigilance and facilitates rapid responses to unexpected visual signals, whereas both excessively low and high volumes weaken alertness and slow reaction speed. Therefore, selecting an appropriate volume range is essential for leveraging music to enhance vigilance.

In addition to auditory stimulation, olfactory stimulation has also been demonstrated to modulate an individual's state of alertness (Sjörs Dahlman et al., 2024). From a neuroanatomical perspective, the olfactory system uniquely projects directly to limbic and paralimbic regions—such as the amygdala, piriform cortex, and orbitofrontal cortex—that are crucial for emotional regulation and sustained attention (Vedaei et al., 2017). Moreover, olfactory stimuli can also activate the trigeminal system, which shares pathways with the olfactory nerve and directly stimulates brainstem arousal centers, thereby enhancing alertness (Jiang et al., 2024). For instance, research by Hoult et al. (2019) provides clearer evidence that peppermint odor can enhance alertness and cognitive performance while simultaneously reducing fatigue. Similarly, Shimizu et al. (2008) found that the application of lavender helps sustain continuous attention during prolonged tasks. It is noteworthy that the concentration of an odor may also significantly influence its regulatory effect. Liu and Hsieh (2018) discovered that low concentrations of lavender aroma can alleviate anxiety, whereas high concentrations improve attentiveness, indicating that aromatherapy at different concentrations exerts differentiated psychological and cognitive regulatory effects. Variations in odor concentration may thus lead to distinctly different intervention outcomes. The impact of concentration differences on vigilance is also reflected in driving research. Huo et al. (2025), in their study on the effects of low, medium, and high concentrations on driving fatigue, found that medium concentration yielded optimal physiological arousal effects based on physiological indicators, although subjective preference leaned toward higher concentrations. Furthermore, Shi et al. (2025) revealed that low-concentration odors tend to induce relaxation, while high concentrations may enhance alertness. However, the specific effects vary depending on the odor type; for example, high-concentration lemon promotes relaxation, whereas high-concentration rosemary and peppermint enhance vigilance. These findings underscore the important role of concentration in modulating alertness.

From a multisensory integration perspective, the interaction between auditory and olfactory modalities is attracting increasing research attention (Li et al., 2025a; Stein and Meredith, 1993). Multisensory integration occurs in structures such as the superior colliculus, where inputs from different senses converge on single neurons, leading to response enhancement when stimuli are spatially and temporally aligned (Stein and Meredith, 1993). Studies indicate that sound and odor can not only mutually enhance or diminish each other's perceived intensity but also jointly influence environmental comfort and overall evaluation (Ba and Kang, 2019a). Research by Bluyssen et al. (2025) found that as traffic noise levels increased, participants perceived the air as more unpleasant and less acceptable, demonstrating a significant cross-modal influence of sound on odor perception. However, not all sensory interactions exacerbate negative experiences. Ba and Kang (2019b) discovered that pleasant fragrances could mitigate the annoyance caused by traffic noise, and combined auditory-olfactory stimulation might be more effective than unimodal interventions in promoting stress recovery. These findings align with recent evidence on the role of color in multisensory contexts, where visual stimuli such as color have been shown to modulate taste expectations, emotional responses, and even olfactory judgments (Bortolotti et al., 2025).

Following the clarification of the independent and interactive effects of volume and concentration on vigilance, the presentation mode of stimuli emerges as another critical yet often overlooked dimension. Existing research has predominantly focused on fixed-intensity sensory stimuli, while the impact of dynamically changing stimulus patterns on alertness states and their sustainability remains underexplored. Mechanistically, constant stimuli may aid initial attention focus, but prolonged exposure tends to induce sensory adaptation (Mackworth, 1968; Jefferis et al., 2024), whereby the sensory system's response gradually diminishes to a sustained, unchanging stimulus (Jing, 2024). This phenomenon has been validated across other sensory modalities. For instance, in the visual domain, Li et al. (2025b) found that dynamically varying lighting patterns were more effective in enhancing individual alertness and task performance compared to constant illumination, a benefit attributed to the delay of sensory adaptation and maintenance of neuromodulatory arousal. Similarly, in auditory and olfactory contexts, fixed stimuli are prone to induce perceptual adaptation and reduced physiological arousal, thereby compromising vigilance maintenance. Functional magnetic resonance imaging (fMRI) studies have provided neuroimaging evidence for this adaptation effect in the olfactory system. For example, Vedaei et al. (2017) observed that while olfactory stimulation activates primary and secondary olfactory regions—including the piriform cortex, amygdala, and insula—the primary olfactory cortex (POC) exhibits a rapid habituation response, with activation signals returning to baseline after the initial 10–15 s of exposure to a constant odor. This neural habituation underscores the limitation of fixed-intensity stimuli in sustaining sensory system engagement (Vedaei et al., 2017). Ma et al. (2026) demonstrated that dynamically increasing concentrations of peppermint odor were more effective in alleviating driving fatigue among young drivers than a constant concentration, with the effect ascribed to the delayed olfactory adaptation afforded by dynamic adjustment. A parallel phenomenon is observed in neurostimulation: Coltman and Hu (2025) reported that constant transcutaneous electrical stimulation led to sensory habituation, whereas a dynamic pattern alternating between stimulation and intervals delayed habituation and maintained more stable sensory feedback. This mechanism is likely applicable to auditory and olfactory stimulation as well; dynamically varying patterns may counteract sensory adaptation by reactivating sensory pathways and delaying neural fatigue and habituation, thereby contributing to the sustained maintenance of appropriate vigilance levels. Furthermore, from the perspective of arousal theory, an individual's vigilance level exhibits a classic “inverted-U-shaped” relationship with the intensity of external stimuli (Yerkes and Dodson, 1908). Moderate stimulus levels are most conducive to maintaining optimal cognitive performance and vigilance; stimuli that are too low or too high may lead to under-arousal or over-arousal, respectively, thereby impairing task performance (Yerkes and Dodson, 1908; Esterman and Rothlein, 2019). A stimulus of fixed intensity, even if initially within the optimal range, may become subjectively perceived as insufficiently arousing due to sensory adaptation or may no longer be optimal as task demands change. Therefore, when investigating the effects of sensory stimuli on vigilance, the presentation mode constitutes a significant moderating factor alongside intensity. Grounded in sensory adaptation theory and the Yerkes–Dodson law, the dynamic incremental mode represents a dual strategy aimed at simultaneously countering sensory adaptation and optimizing arousal levels. By gradually and controllably increasing stimulus intensity in stages, it seeks to break the adaptation cycle and maintain the novelty of sensory input; it dynamically keeps an individual's perceptual and physiological arousal levels within the peak region of the “inverted-U-shaped” curve—the optimal performance zone.

In summary, in safety-critical domains, the maintenance and enhancement of vigilance are of great significance. Auditory (music) and olfactory (odor) stimuli have been proven to have the potential to modulate alertness, with volume and concentration being key parameters influencing their effectiveness. Furthermore, multisensory integration research indicates that auditory and olfactory inputs interact, collectively shaping an individual's cognitive and emotional states. However, existing studies have predominantly focused on stimuli of fixed intensity, paying less attention to the influence of stimulus presentation mode. Given that constant stimulation is prone to inducing sensory adaptation, dynamically varying patterns of stimulation may sustain vigilance levels more effectively by delaying the adaptation process. Nevertheless, there is currently a lack of systematic comparisons, within a unified experimental design, of the effects of auditory and olfactory modalities under constant vs. incremental modes, and few studies have explored whether dynamic bimodal combinations can produce additive benefits in vigilance maintenance. Therefore, this study aims to investigate the effects of music volume and odor concentration on vigilance under both constant and incremental presentation modes. Compared to the constant mode, the incremental mode is expected to enhance vigilance levels more effectively within a single modality, while the dual-incremental condition may yield the most significant improvement, reflecting the additive advantage of cross-modal dynamic stimulation in sustaining vigilance. The findings are expected to provide empirical evidence for optimizing and innovating multisensory vigilance intervention strategies.

## 2 Hypotheses

Previous research has demonstrated that both auditory (music) and olfactory (odor) stimuli can effectively modulate an individual's state of vigilance. However, stimuli of constant intensity are prone to induce sensory adaptation, leading to a gradual decline in vigilance over the course of a task. In contrast, dynamically varying stimulus presentation modes, such as an incremental mode, may more effectively counteract vigilance decrement during prolonged tasks by delaying sensory adaptation and maintaining neuromodulatory arousal levels. Furthermore, incremental input from both modalities could produce a vigilance-enhancing effect that surpasses the sum of their individual contributions through cross-channel integration. Therefore, the presentation mode of music volume and odor concentration is hypothesized to influence overall vigilance performance and its temporal trajectory during the task. The hypotheses are as follows:

H1: Compared to the blank control condition (no music, no odor), any experimental condition containing either music or odor stimulation will result in better vigilance.
H2: The incremental mode will be more effective than the constant mode in enhancing vigilance, both for music volume (H2a) and for odor concentration (H2b).
H3: Vigilance performance benefits additively from the incremental presentation of music and odor and is highest when both modalities are presented in the incremental mode.

## 3 Methodology

### 3.1 Experimental design

This study employed a within-subjects design, wherein all participants were exposed to every experimental condition. This approach was adopted to control for the influence of individual differences on vigilance performance. The core experiment followed a 2 × 2 factorial design, complemented by a blank control condition (no music, no odor) serving as a baseline reference. All sessions were conducted under controlled environmental settings. Each participant completed all five conditions across different time periods. The use of a within-subjects design (Morales et al., 2017), combined with a Latin square design (Li et al., 2019) to counterbalance the order of conditions across participants, effectively controlled for inter-individual variability, increased statistical power, and minimized potential biases arising from order effects. The 26 participants were randomly assigned to the five sequences generated by the Latin square, with five sequences assigned to five participants each and one sequence assigned to six participants, ensuring a near-balanced distribution. These measures collectively enhanced the internal validity of the experimental findings.

To assess participants' vigilance states under different experimental conditions, this study utilized the Psychomotor Vigilance Task (PVT) as the primary behavioral measurement tool. The PVT is a widely used sustained-attention test paradigm that quantifies alertness by measuring reaction times to visual stimuli presented at random intervals (Dinges and Powell, 1985). It has been proven by multiple studies to be an effective tool for evaluating changes in vigilance, demonstrating high sensitivity and ecological validity, particularly in multisensory integration research (Wang et al., 2024). In each experimental condition, participants were required to complete a standardized PVT session, and their performance was used for subsequent statistical analysis.

Music with a mild melody and steady rhythm was selected as the auditory stimulus, focusing on the effect of volume variation on vigilance. The constant volume was set at 60 dB, a level supported by Mizoguchi and Tsugawa (2012), who found that 63 dB was most conducive to safe driving and sustained driving alertness. To maintain consistency, the medium volume level in the dynamic incremental mode was also set at 60 dB. Following Ma et al., who used 50 dB as a low loudness level, and based on Yang et al. (2022), whose research showed that light rock music at 45–50 dB significantly improved laparoscopic operation accuracy and alertness in novice surgeons, the low volume was set at 50 dB. Bishop et al. (2009) reported that participants listening to higher-volume music (75 dBA) showed significantly faster reaction times in choice reaction tasks, while Ma et al. (2014) found that excessively high volumes (80 dB and 95 dB) impaired alertness. Therefore, the high volume was set at 70 dB. The music volume settings were thus defined as constant volume at 60 dB and incremental volume at 50 dB, 60 dB, and 70 dB.

Peppermint essential oil was selected as the olfactory stimulus due to its well-documented alertness-enhancing effects demonstrated in multiple studies (Jiang et al., 2024). The primary active component in peppermint oil, menthol, possesses confirmed neurostimulatory properties. It can directly activate the central nervous system via the trigeminal nerve pathway, thereby enhancing alertness and cognitive performance (Jiang et al., 2024). Systematic reviews by Hoult et al. (2019) and Jiang et al. (2024) highlight the significant efficacy of peppermint in countering fatigue and promoting mental refreshment. Research by Hirata (2001) on peppermint odor release at concentrations of 1, 10, and 100% identified 10% as the optimal concentration for enhancing alertness. Scholar Tang (2022) further refined the concentration gradation to 5, 10, and 15%, confirming that a 10% concentration yields the optimal short-term fatigue-alleviating effect, although its efficacy exhibits a time-dependent decay, and the optimal release duration varies across concentrations. Since most scholarly evidence points to 10% as the optimal concentration for peppermint odor release, the constant concentration was set at 10%. Consequently, the medium concentration for both the constant and incremental concentration adjustment modes in this experiment was established at 10%. However, existing literature shows variation in the demarcation of low and high concentrations. According to Tang et al. (2022), high concentrations of peppermint can significantly improve fatigue-alleviation effects. Although that study did not disclose specific compositions or ratios, and its concentration classification differs from other research, it nevertheless indicates the potential of high-concentration odors in mitigating fatigue. To establish a more scientifically grounded acceptability range for different concentrations, this study referenced the non-acceptance rate criteria employed by Mitsuda et al. (2024) in odor acceptability evaluations. Using 15, 20, and 30% as thresholds corresponding to high cleanliness requirements, recommended values, and permissible limits, respectively, 30% was adopted as the permissible limit for odor acceptance in this context. On the other hand, research by Jin et al. (2018) suggests that when concentration reaches 100 ppm, strongly stimulating odors can shift an individual from a calm to an excited state, demonstrating the significant impact of low-concentration odors on alertness. Hirata (2001) found that a 1% concentration can enhance alertness through olfactory transmission. Therefore, this study set the low concentration at 1% and the high concentration at 30%. The odor concentration was thus defined as a constant concentration of 10% and an incremental concentration (1, 10, and 30%). In the incremental mode, the stimulus intensity was increased stepwise in three consecutive stages, each lasting 3 min and 20 s, precisely synchronized with the three blocks of the PVT task. Specifically, during the first block, the music volume was maintained at 50 dB and the odor concentration at 1%; during the second block, they were raised to 60 dB and 10%, respectively; and during the final block, they were further increased to 70 dB and 30%.

### 3.2 Participants

An a priori power analysis was performed using G^*^Power 3.1 software to determine the minimum sample size required for detecting a medium effect size. Based on Cohen's definition of effect size, the analysis was conducted with the following parameters: significance level (α) = 0.05, statistical power (1 – β) = 0.8, effect size f^*^ = 0.25 (corresponding to a medium effect according to Cohen's conventions, with small and large effects defined as f = 0.10 and f = 0.40, respectively), assuming a high correlation (*r* = 0.5) among repeated measures (Cohen, 2013). The calculation, using repeated-measures ANOVA with nonsphericity correction (ε = 1), yielded a theoretical minimum sample size of 21 participants. To enhance the study's tolerance to data variability and the robustness of the findings, a total of 26 participants (aged 20–26 years) were ultimately recruited. The sample included 13 males (Mean = 23.8, SD = 2.15) and 13 females (Mean = 24.4, SD = 2.98). All participants reported normal hearing and olfactory function and had no history of neurological or psychiatric disorders. To minimize the potential influence of professional background on music perception, individuals with formal music training were excluded.

In this study, formal music training was operationalized as cumulative, systematic musical instruction (including but not limited to instrumental performance, music theory, and solfège) lasting 1 week or more. This exclusion criterion was applied to reduce the likelihood that specialized auditory expertise or familiarity with musical structure would confound the effects of the volume manipulation, ensuring that the observed results reflect general population responses rather than trained auditory processing. To further control for physiological factors known to influence olfactory sensitivity, female participants were scheduled outside of their menstrual period (Doty et al., 1981). Participants were required to abstain from caffeine and alcohol for 24 h prior to the experiment and to maintain regular sleep duration. Written informed consent was obtained from all participants before the experiment, and they were informed of their right to withdraw at any time without penalty. Appropriate compensation was provided upon completion. The study protocol was approved by the Medical Ethics Committee of Hebei Medical University (Approval No.: 2025-130). All procedures strictly followed the ethical principles of the Declaration of Helsinki.

All participants self-reported normal hearing and olfactory function. To further control for individual differences in responses to olfactory stimuli, a brief screening of olfactory sensitivity was conducted prior to the formal experiment. This test required participants to identify a series of peppermint odors at different concentrations. Only participants who could correctly identify the standard concentration of 10% were included. All participants passed this screening, indicating they possessed normal olfactory perception abilities. Furthermore, to isolate potential interference from musical style, several measures were taken. First, participants were not informed about the specific music piece in advance; they were only told that there would be background sound. During the practice session before the formal experiment, participants practiced the tasks with the same background music, ensuring preliminary adaptation to the music itself. This was intended to minimize additional influences arising from unfamiliarity, initial curiosity, or aversion to the music. Second, during the experimental instructions, participants were guided to focus their attention on the task itself rather than on the music content and were informed that the music was merely a part of the background environment. Through these methods, we aimed to reduce extraneous variable interference in both the auditory and olfactory dimensions. This ensured that the effects observed in the experiment primarily stemmed from the interaction of the olfactory stimuli and the auditory background, rather than from individual preferences for specific scents or musical styles, or emotional reactions thereto.

### 3.3 Stimuli

#### 3.3.1 Music

In this study, the song Starin' Through My Rear View was chosen as the auditory stimulus. This piece is representative of the West Coast Hip Hop/Gangsta Rap genre. It is important to acknowledge that, unlike instrumentals, this genre often carries complex lyrical narratives and socio-political commentary. The piece belongs to the country rock genre and is characterized by a mild melody and a steady rhythm, with a tempo of approximately 98 BPM and major mode. Importantly, the stable rhythmic structure ensures that the volume manipulation is not confounded by abrupt changes in rhythmic complexity (Wang et al., 2024). The music stimulus was delivered via high-fidelity over-ear headphones to ensure all participants received a consistent average sound intensity. During the experiment, the volume was manipulated according to two designed modes: constant mode and incremental mode. In the constant mode, the average music volume was maintained at 60 dB. In the incremental mode, the volume was increased from 50 dB to 60 dB and finally to 70 dB during the task period. The duration of each volume level was equal to ensure a smooth and controlled incremental process. Importantly, participants were not informed when the volume transitions occurred, preventing them from anticipating the changes in volume level between blocks.

Given that the song contains lyrics, we took several steps to minimize any potential influence of semantic content on participants' cognitive or emotional states. Participants were not informed of the song's title or theme beforehand; they were simply told that background audio would be present. During the practice session, they were exposed to the music to reduce novelty or initial emotional reactions. Moreover, the instructions explicitly directed participants to focus on the PVT and treat the music as part of the ambient environment, thereby encouraging them to process it as background rather than as a semantic stimulus.

#### 3.3.2 Odor

In this study, AASkincare peppermint essential oil was used as the olfactory stimulus material to ensure ingredient consistency and experimental controllability. The odor was delivered via a mini electronic aroma diffuser, which precisely controlled the volatilization rate and concentration of the essential oil, preventing odor dispersion into the experimental environment and affecting other equipment or participants. The concentration settings were based on previous research. The constant concentration was set at 10%. The incremental concentration mode was set at 1, 10, and 30%, corresponding to low, medium, and high concentration levels, respectively. The duration of each concentration level in the incremental mode was matched with the corresponding stage in the music volume increment, ensuring temporal synchronization of the bimodal stimuli. To minimize the impact of environmental diffusion, the diffuser was placed at a fixed distance and position relative to the participant, and all sessions were conducted in the same room with stable ventilation conditions. Participants were instructed to maintain a natural and regular breathing pattern and to keep a consistent seated posture throughout each experimental session.

### 3.4 Experimental settings

**Figure 1:**
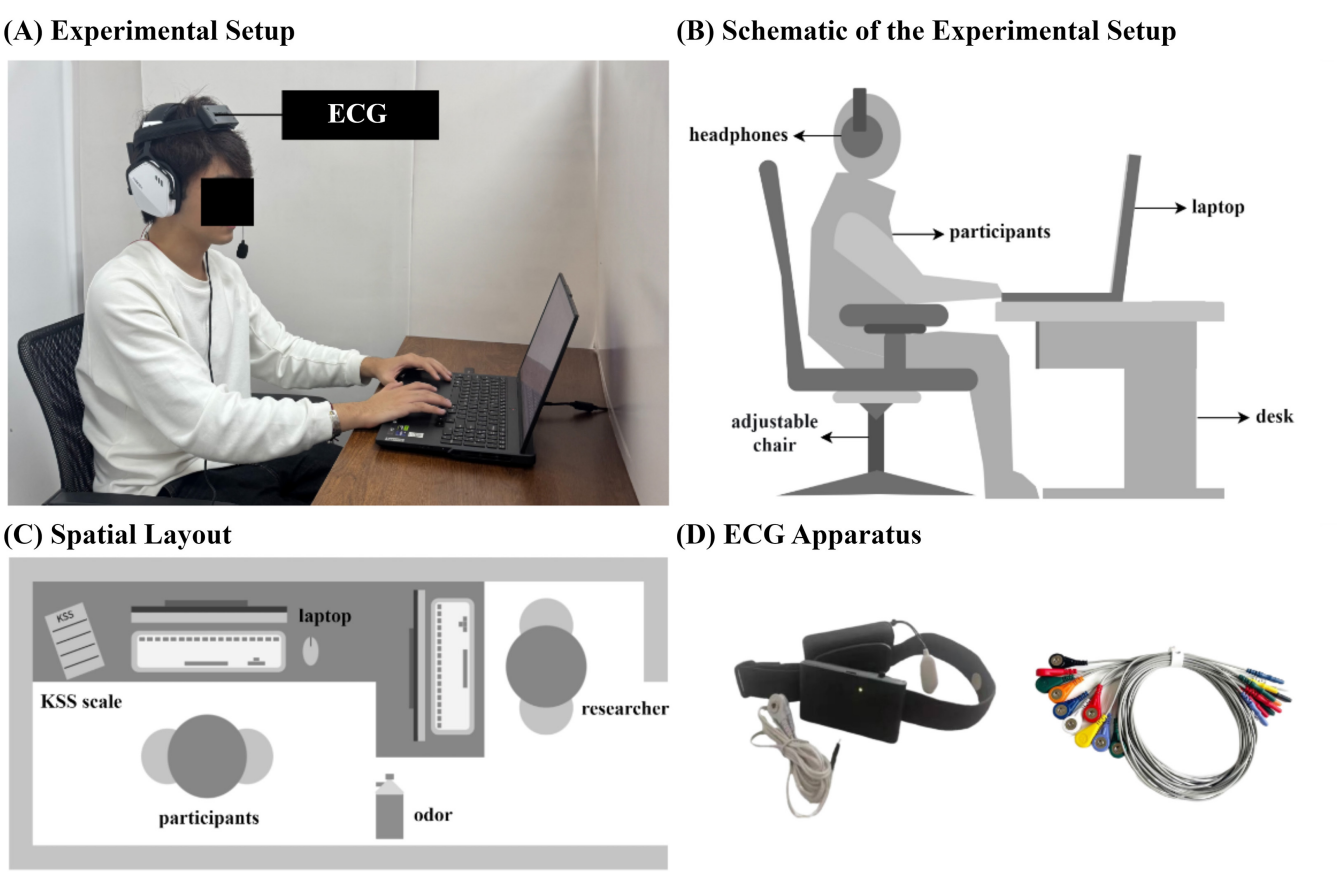
Laboratory setup and equipment layout diagram [**(A)**, Experimental Setup; **(B)**, Schematic of the Experimental Setup; **(C)**, Spatial Layout; **(D)**, ECG Apparatus].

The experiment was conducted in a human factors laboratory. Doors and windows were closed during the experiment to control ambient noise. A Lenovo laptop (model Y9000P, 16-inch screen, resolution 2,560 × 1,600) was used to present the PVT, to ensure precise temporal presentation of the visual stimulus, the display was configured to its maximum refresh rate of 240 Hz, yielding an inter-frame interval of approximately 4 ms. Consumer-grade LCD monitors, when suitably configured, are capable of the high temporal precision required for vision science and vigilance research (Zhang et al., 2018). To ensure the reproducibility of the findings and facilitate cross-study comparisons in future research, the present study adhered to current methodological recommendations by providing a detailed account of the experimental environment (Lin et al., 2023). Specifically, the visual stimuli consisted of a white background (luminance ≈ 120 cd/m^2^) and a black millisecond counter (luminance < 1 cd/m^2^). Ambient illumination was maintained at approximately 300 lx at eye level using fluorescent ceiling lights. All sessions were conducted under identical lighting conditions, and the display luminance and contrast settings were kept constant across all experimental sessions to ensure internal consistency across participants and conditions. Participants were seated on an adjustable chair, with their eye level aligned horizontally with the screen to facilitate keyboard responses. Electrocardiogram (ECG) data were continuously recorded using a multimodal human factors experimental device and its accompanying system produced by Beijing Langxin Renyin Technology Co., Ltd. (Haidian, Beijing), which monitored participants' ECG activity in real time. During the experiment, participants wore headphones to receive auditory (music) stimulation and received olfactory stimulation via an electronic aroma diffuser. All environmental conditions were kept consistent across sessions. The specific setup is illustrated in Figure 1.

### 3.5 Measurements

#### 3.5.1 Subjective measures

Alertness assessment methods can be divided into subjective and objective detection. Subjective detection primarily utilizes scale ratings, such as the Karolinska Sleepiness Scale (KSS) (Åkerstedt and Gillberg, 1990), the Toronto Hospital Alertness Test (THAT), the Mindful Attention Awareness Scale (MAAS), and the Attentional Control Scale (ACS), among others (Prendez et al., 2026). Subjective scales can effectively reflect an individual's internal perception of their own alertness state. The significant correlation between psychological subjective ratings under experimental conditions, PVT behavioral performance, and physiological indicators demonstrates that such scales not only reflect internal perception but are also consistent with external behavior and physiological state. To comprehensively assess changes in participants' subjective alertness under sensory stimulation, this study selected the Karolinska Sleepiness Scale (KSS) as the primary subjective measurement tool. The KSS is a concise and efficient single-item scale that requires participants to self-rate their current level of sleepiness on a nine-point scale (1 = extremely alert, 9 = extremely sleepy) (Åkerstedt and Gillberg, 1990). Widely used in sleep and alertness research, the KSS is regarded as an important standard for assessing subjective alertness (Marois et al., 2023). Its structure, ranging from extreme alertness to extreme sleepiness, essentially reflects a continuum of alertness, with low scores corresponding to high alertness and high scores corresponding to low alertness. In attention tasks and multisensory integration research, the KSS has been extensively employed as an effective tool for assessing subjective alertness, demonstrating good reliability, validity, and ecological validity (Laharnar et al., 2020). Therefore, the KSS was adopted as the measurement tool for subjective alertness in this study.

#### 3.5.2 Physiological measurements

To objectively assess physiological changes in alertness, Heart Rate Variability (HRV), which reflects the variation in consecutive heartbeat intervals, serves as an important indicator of autonomic nervous system function. Common HRV metrics include time-domain indices (e.g., SDNN, RMSSD) and frequency-domain indices (e.g., LF, HF). Among these, both SDNN and HF have significant associations with alertness. SDNN reflects the overall regulatory capacity of the autonomic nervous system and is related to bodily adaptation and arousal levels (Wang et al., 2023). HF primarily represents parasympathetic nervous activity, which is closely linked to attentional focus and psychological relaxation (Zhou and Zhou, 2022). Existing research indicates that in sustained attention tasks, HF is a more sensitive and stable physiological correlate of alertness changes. When alertness increases, HF typically shows a moderate decrease, suggesting the inhibition of parasympathetic activity and a shift toward attentional concentration. Conversely, when alertness declines, HF tends to increase (Vicente et al., 2016). Therefore, although both SDNN and HF can reflect aspects of alertness modulation, HF corresponds more directly to the autonomic dynamics associated with alertness changes. Consequently, HF was selected as the primary physiological dependent variable in this study, aiming to provide objective physiological support for the behavioral findings. However, it should be noted that the relationship between HF and alertness is not univocal; its variations are also modulated by factors such as breathing patterns, task types, and individual differences (Bernardi et al., 2000), leading to some inconsistencies across studies. For instance, while many studies report that heightened alertness during sustained attention tasks is associated with reduced HF due to parasympathetic withdrawal (Zhou and Zhou, 2022; Vicente et al., 2016; Yuda et al., 2017), other studies have observed that under certain conditions, such as during magnetic stimulation, heightened alertness may be accompanied by increased HF (Cui et al., 2017). These divergent findings suggest that the autonomic response to increased vigilance is not uniform; it depends on the specific engagement of neural circuits and the balance between sympathetic and parasympathetic outflows (Zhou and Zhou, 2022; Cui et al., 2017). In tasks that demand sustained attention to external stimuli with minimal cognitive load—such as the PVT—enhanced vigilance is typically achieved through active inhibition of parasympathetic activity, promoting sympathetic dominance and enabling rapid behavioral responses. This pattern is consistently reflected in reduced HF (Zhou and Zhou, 2022). In the present study, the PVT is a monotonous, low-load task requiring sustained alertness to infrequent visual stimuli without complex cognitive demands. This autonomic profile aligns with findings from similar paradigms, where reduced HF is consistently observed (Zhou and Zhou, 2022; Vicente et al., 2016; Yuda et al., 2017). Participants were instructed to maintain regular breathing throughout each session to minimize respiration-related confounds (Bernardi et al., 2000). Therefore, the decrease in HF under incremental stimulation conditions can be reasonably interpreted as reflecting enhanced physiological arousal and improved vigilance. Thus, HF was employed as the primary physiological indicator to capture autonomic trends across experimental conditions. Integrating HF with subjective scales and behavioral performance allows for a more comprehensive analysis and discussion of the experimental results.

According to the recommendations of the (Task Force of the European Society of Cardiology the North American Society of Pacing Electrophysiology, 1996), the standard recording duration for heart rate variability (HRV) analysis is typically 5 min to ensure the stability and comparability of frequency-domain indicators (Task Force of the European Society of Cardiology the North American Society of Pacing Electrophysiology, 1996). However, an increasing number of recent studies have explored the feasibility of short-term HRV analysis in dynamic monitoring and real-time assessment. For instance, Wang et al. (2023) explicitly stated in their study that “5 min is the international standard duration for HRV analysis,” but to achieve the research objective of “real-time assessment,” they adopted a 1-min analysis window and validated its effectiveness in practical applications. Furthermore, numerous contemporary studies have employed HRV recording windows shorter than 5 min under similar temporal constraints, yielding reliable physiological responses in domains such as alertness, cognitive load, and fatigue monitoring (Wang et al., 2023; Lee et al., 2022; Shaffer et al., 2020). Therefore, although the HRV segment collected in each experimental condition of this study was 3 min and 20 s—slightly shorter than the traditional standard duration—this recording length still possesses adequate physiological measurement validity and experimental rationale, considering the continuity requirements of the experimental design, the natural segmentation of the task structure (synchronized with the three blocks of the PVT task), and the broad applicability of short-term HRV in dynamic contexts.

#### 3.5.3 Psychomotor Vigilance Task

**Figure 2:**
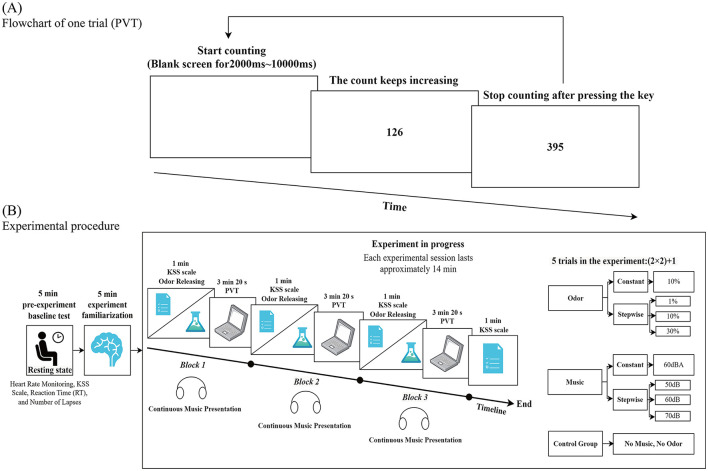
Schematic diagram of the Psychomotor Vigilance Task (PVT) experimental paradigm [**(A)** = Flowchart of one trial (PVT); **(B)** = Experimental procedure].

The PVT was employed as the primary behavioral measurement tool in this study (Li et al., 2024; Kouba et al., 2023). The task was programmed using PsychoPy software and presented on a Lenovo laptop (model Y9000P) with a 16-inch screen, which served as the experimental apparatus for all sessions. To ensure high temporal precision of the visual stimuli, the display was configured to its maximum refresh rate of 240 Hz, yielding an inter-frame interval of approximately 4 ms. Responses were recorded via the built-in laptop keyboard. While recent benchmark data indicate that consumer-grade keyboards can introduce mean response delays ranging from approximately 11 ms to over 70 ms, depending on the model and connection type (Tang et al., 2025), such absolute delays do not systematically affect the relative differences between experimental conditions, thereby preserving the validity of within-subject comparisons. A visual stimulus appeared at the center of a white-background screen at random inter-stimulus intervals (2–10 s). Participants were required to press the spacebar as quickly as possible upon stimulus onset, as detailed in Figure 2A. The system automatically recorded the RT for each response. A lapse was recorded if the RT exceeded 500 ms. The task duration was set to 10 min to ensure adequate capture of alertness dynamics. Consequently, this study selected mean RT (excluding data where RT was less than 100 ms) and the number of lapses (trials with RT > 500 ms) as the core behavioral metrics from the PVT task to quantify participants' alertness levels (Wang et al., 2024). Previous research has indicated that a 10-min PVT session is sufficient to effectively capture the initial decline in alertness, being particularly sensitive to sensory adaptation and early fatigue responses (Jung et al., 2011; Grant et al., 2017). Furthermore, a relatively short PVT duration helps control for cumulative fatigue effects within the within-subjects design, ensuring participants maintain stable attention levels across different experimental conditions and avoiding potential learning effects or excessive fatigue from prolonged tasks that might impact performance in subsequent sessions. Therefore, the 10-min PVT duration was deemed adequate to demonstrate the advantage of the incremental mode over the constant mode in mitigating early alertness decline.

### 3.6 Experimental procedure

Prior to the experiment, all equipment was calibrated and tested to ensure the accuracy of the data. The output music volume from the headphones and the electronic aroma diffuser was adjusted and verified to meet the preset requirements. Upon arrival at the laboratory, participants first completed a basic information form, and the experimenter then provided a detailed explanation of the experiment's purpose, procedures, and precautions. Written informed consent was obtained from all participants before proceeding to the experimental session.

Baseline measurements were conducted before the formal experiment began. Prior to baseline measurements, participants were instructed to sit quietly and breathe naturally and steadily for 2 min to stabilize their respiratory pattern. This procedure was implemented to minimize the potential confounding effect of respiratory variations on heart rate variability (HRV), particularly the high-frequency component (HF), which is known to be sensitive to changes in breathing frequency. Participants were reminded to maintain a regular breathing rhythm throughout each experimental session. Participants sat quietly and rested for 5 min, during which their electrocardiogram (ECG) signals were recorded as the physiological baseline for HRV. Following this, participants completed the Karolinska Sleepiness Scale (KSS) to assess their initial alertness state. Subsequently, participants performed a practice session of the PVT lasting approximately 5 min to familiarize themselves with the task operation. The formal experiment employed a within-subjects design, comprising five experimental conditions: four combinations of music and odor conditions and one no-music, no-odor blank control condition. All conditions were counterbalanced across participants using a Latin square design (Li et al., 2019). A rest period of at least 24 h was implemented between each condition to mitigate fatigue effects.

The experimental procedure for each condition was as follows: According to the condition settings, background music was played through headphones, and odor was released via an aroma diffuser. In the constant mode, the volume or concentration was maintained at a fixed level. In the incremental mode, the volume or concentration was gradually increased in predefined stages during the task.

(1) Following a 1-min odor release, participants immediately began a 3-min 20-s PVT task, with music playing continuously. Visual stimuli appeared at the center of the screen at random inter-stimulus intervals (ranging from 2 to 10 s). Participants were required to press the response key as quickly as possible upon stimulus onset. Reaction time (RT) and the number of lapses were automatically recorded by the system. Simultaneously, ECG data for HRV analysis were continuously collected. After completing the first block of the PVT task, participants underwent a subjective assessment, filling out the KSS to report their current subjective alertness level.

(2) After another 1-min odor release, participants immediately began the second 3-min 20-s PVT block, with music continuing to play. Upon completion of the second PVT block, a subjective assessment was conducted again using the KSS.

(3) Following a third 1-min odor release, participants began the final 3-min 20-s PVT block, with music still playing continuously. After the third PVT block concluded, a final subjective assessment was performed using the KSS.

This concluded one complete experimental session for that specific condition. Each session was followed by a 24-h rest period before the participant proceeded to the next session. Each session followed the aforementioned sequence of PVT tasks and KSS assessments. The order of the five experimental sessions was randomized for each participant. After completing all sessions, participants were thanked for their participation and engaged in a brief interview about their experimental experience. The detailed procedure is illustrated in Figure 2B.

## 4 Results

### 4.1 Reliability

Experimental data were initially collected from 26 participants. Data from one participant were excluded due to displacement of the worn devices during the procedure. Additionally, data from another participant, which exhibited substantial disruption, were removed during the preprocessing stage after outlier detection. Ultimately, valid data from 24 participants were retained. Subjective alertness was assessed using the Karolinska Sleepiness Scale (KSS). Physiological alertness was indexed by high-frequency heart rate variability (HF), recorded via a heart rate monitoring device. Behavioral alertness was measured using the PVT, with reaction time (RT) and number of lapses recorded. The reliability and validity of the data were subsequently evaluated using SPSS Statistics version 27.

Reliability test: The amount of valid data for this experiment was small, considering the duration and cost of the experiment. To reflect the stability and consistency of the experimental data, a reliability analysis was performed before data analysis. As Cronbach's α, a reliability coefficient, is influenced by sample size and the number of items, SPSS Statistics 27 was employed for the analysis. The KSS yielded a Cronbach's α of 0.841. In exploratory research, a Cronbach's α value exceeding 0.8 is considered to indicate good reliability, supporting the dependability of the collected subjective data.

Validity test: To assess data suitability and analysis feasibility, the Kaiser-Meyer-Olkin (KMO) test was conducted to measure sampling adequacy alongside Bartlett's Test of Sphericity. The KMO values range from 0 to 1. It is generally accepted that a KMO value greater than 0.6 indicates that the data have a certain degree of sampling adequacy and are suitable for further analysis. The KMO value of the KSS scores was 0.650, and Bartlett's test of sphericity yielded an approximate chi-square value of 59.324 (*df* = 10; *p* ≤ 0.001)—the correlation matrix significantly deviated from the identity matrix. These results support the suitability of the data for further analysis. Given the small sample size, partial eta-squared was used to measure ES as a reference for validity testing. As an important indicator of the strength of experimental manipulation, ES is not influenced by sample size. In SPSS, the commonly used threshold values for interpreting ES levels—small, medium, and large—based on partial eta-squared are as follows: 0.01 < Partial η^2^ < 0.06 indicates a small ES, 0.06 < Partial η^2^ < 0.14 indicates a medium ES, and Partial η^2^ > 0.14 indicates a large ES, effectively guaranteeing the validity of further analysis.

All collected data were statistically analyzed using SPSS 27. A Shapiro–Wilk test of normality was performed on the main dependent variables (KSS scores, RT, number of lapses, and HF). The results indicate that the residuals of the data under all conditions conformed to a normal distribution (all *p* > 0.05).

### 4.2 Subjective alertness (KSS)

#### 4.2.1 Mean KSS scores

**Figure 3:**
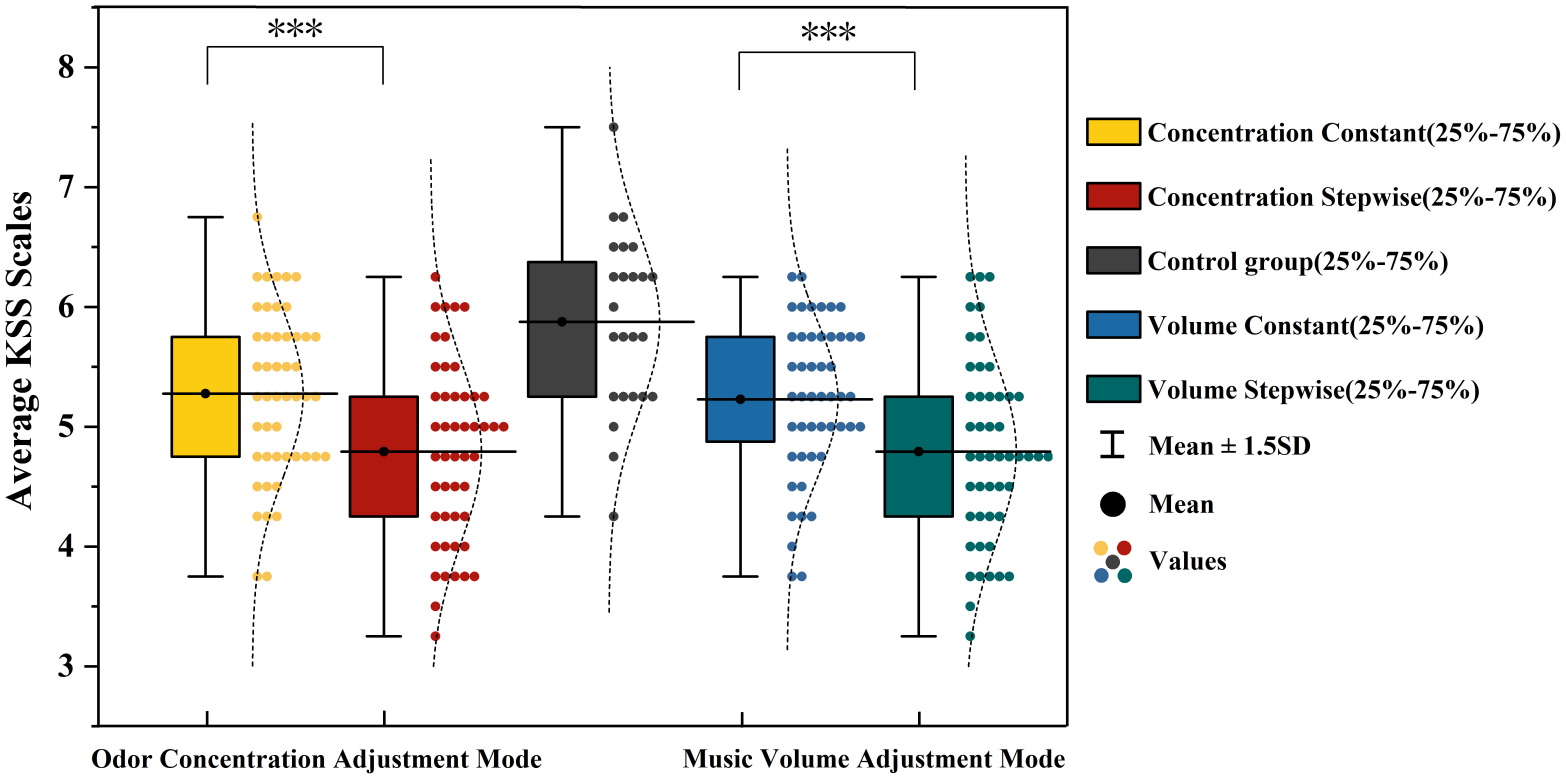
Analysis results of mean KSS scores under different music volume and odor concentration adjustment modes. ***represents *p* < 0.001.

The subjective alertness of participants was assessed using the Karolinska Sleepiness Scale (KSS). To examine the effects of music volume adjustment mode and odor concentration adjustment mode on subjective alertness, a repeated-measures ANOVA was conducted, with the no-music, no-odor control group serving as the baseline (M = 5.87, SD = 0.74). The ANOVA results are presented in Table 1. The main effect of odor concentration adjustment mode was significant, *F*_(1, 23)_ = 18.964, *p* < 0.001, η*p*^2^ = 0.452. KSS scores under the incremental concentration condition (M = 4.78, SD = 0.73) were lower than those under the constant concentration condition (M = 5.24, SD = 0.66). The main effect of music volume adjustment mode was also significant, *F*_(1, 23)_ = 26.008, *p* < 0.001, η*p*^2^ = 0.531, with lower KSS scores in the incremental volume condition (M = 4.79, SD = 0.76) compared to the constant volume condition (M = 5.23, SD = 0.64). Pairwise comparisons revealed that the control group yielded higher KSS scores than all experimental conditions (all *p* < 0.001). Moreover, the dual-incremental condition (incremental music and incremental odor) produced lower KSS scores than the dual-constant condition (*p* < 0.001), the music-incremental-only condition (*p* = 0.017), and the odor-incremental-only condition (*p* = 0.019). The specific comparisons of mean KSS scores across different adjustment modes are illustrated in Figure 3.

**Table 1:** ANOVA results of KSS scores under music volume and odor concentration adjustment modes.

Factor	M	SD	95% CI	*F*	*df*1	*df*2	*p*	*ηp^2^*
**Control group**	5.87	0.74	(5.56, 6.19)					
**Odor concentration adjustment mode**				18.964	1	23	< 0.001^***^	0.452
Concentration constant	5.24	0.66	(5.05, 5.43)					
Concentration stepwise	4.78	0.73	(4.57, 4.99)					
**Music volume adjustment mode**				26.008	1	23	< 0.001^***^	0.531
Volume constant	5.23	0.64	(5.04, 5.42)					
Volume stepwise	4.79	0.76	(4.57, 5.01)					
**Music volume adjustment mode** ^*^odor concentration adjustment mode				0.723	1	23	0.404	0.031

F: M, Mean; SD, Standard Deviation; 95% CI, 95% Confidence Interval; *F*-value; df, degrees of freedom; *p*, significance level; ηp^2^, partial eta-squared effect size; *p*-values are marked as follows:

^*^p < 0.05,

^**^p < 0.01,

^***^p < 0.001.

#### 4.2.2 Temporal changes in mean KSS scores

**Figure 4:**
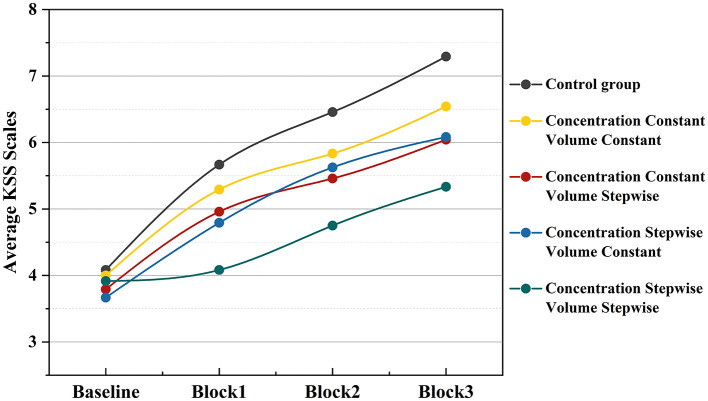
Temporal trend of KSS scores under different experimental conditions.

The temporal trend of subjective KSS scores is illustrated in Figure 4. Mean KSS scores increased progressively across all experimental conditions as the PVT task advanced. Notably, the blank control group exhibited the highest scores at each time point, with a relatively steep upward trend. In contrast, KSS scores under any condition involving auditory (music) or olfactory (odor) stimulation remained consistently lower than those of the control group. Among these, the condition in which both music volume and odor concentration were presented incrementally yielded the lowest KSS scores throughout the task and showed the most gradual increase over time. The score trajectories for the other experimental conditions—such as constant music with constant odor, incremental music only, and incremental odor only—fell between those of the blank control and the dual-incremental group. Although their scores were initially close to those of the dual-incremental group, they increased more markedly as the task progressed, trending toward the pattern observed in the control group.

### 4.3 PVT behavioral results

#### 4.3.1 Mean RT

**Figure 5:**
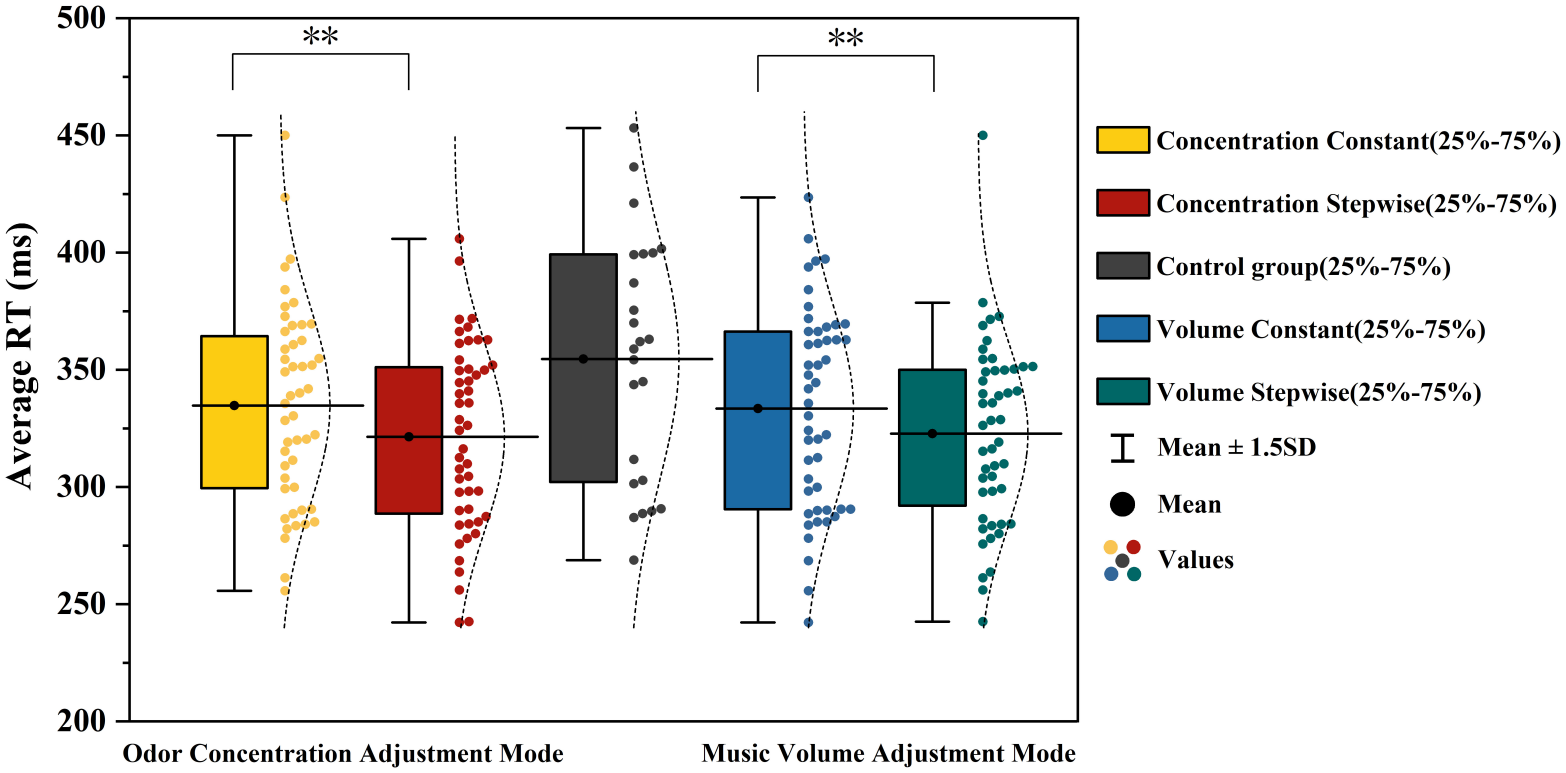
Analysis results of mean RT under different music volume and odor concentration adjustment modes. **represents *p* < 0.01.

The mean reaction time (RT) from the Psychomotor Vigilance Task (PVT) was employed as the core behavioral indicator of participants' alertness. To examine the effects of music volume adjustment mode and odor concentration adjustment mode on behavioral alertness, a repeated-measures ANOVA was conducted, with the no-music, no-odor control group serving as the baseline (M = 354.58 ms, SD = 52.38). The results are presented in Table 2. The ANOVA revealed a significant main effect of odor concentration adjustment mode, *F*_(1, 23)_ = 11.755, *p* = 0.002, η*p*^2^ = 0.338. Mean RT under the incremental concentration condition (M = 321.43 ms, SD = 39.84) was shorter than that under the constant concentration condition (M = 334.70 ms, SD = 42.35). The main effect of music volume adjustment mode was also significant, *F*_(1, 23)_ = 8.645, *p* = 0.007, η*p*^2^ = 0.273, with shorter RT in the incremental volume condition (M = 322.72 ms, SD = 39.33) compared to the constant volume condition (M = 333.41 ms, SD = 43.19). Pairwise comparisons revealed that mean RT was shorter under all experimental conditions compared to the control group (dual-constant: *p* = 0.001; music-incremental-only: *p* = 0.009; odor-incremental-only: *p* = 0.004; dual-incremental: *p* < 0.001). Among these, the dual-incremental condition yielded the shortest mean RT. Further pairwise comparisons showed that mean RT in the dual-incremental condition was significantly shorter than in the dual-constant condition (*p* = 0.006) and the music-incremental-only condition (*p* = 0.040), but the difference from the odor-incremental-only condition did not reach statistical significance (*p* = 0.065). Specific comparisons of mean RT across different adjustment modes are illustrated in Figure 5.

**Table 2:** ANOVA results of mean RT under music volume and odor concentration adjustment modes.

Factor	M	SD	95% CI	*F*	*df*1	*df*2	*p*	*ηp^2^*
**Control group**	354.58	52.38	(332.46, 376.70)					
**Odor concentration adjustment mode**				11.755	1	23	0.002^**^	0.338
Concentration constant	334.70	42.35	(322.40, 346.99)					
Concentration stepwise	321.43	39.84	(309.86, 333.00)					
**Music volume adjustment mode**				8.645	1	23	0.007^**^	0.273
Volume constant	333.41	43.19	(320.87, 345.96)					
Volume stepwise	322.72	39.33	(311.29, 334.14)					
**music volume adjustment mode** ^*^odor concentration adjustment mode				2.058	1	23	0.165	0.031

F: M, Mean; SD, Standard Deviation; 95% CI, 95% Confidence Interval; *F*-value; df, degrees of freedom; *p*, significance level; ηp^2^, partial eta-squared effect size; *p-*values are marked as follows:

^*^p < 0.05,

^**^p < 0.01,

^***^p < 0.001.

#### 4.3.2 Temporal changes in mean RT

**Figure 6:**
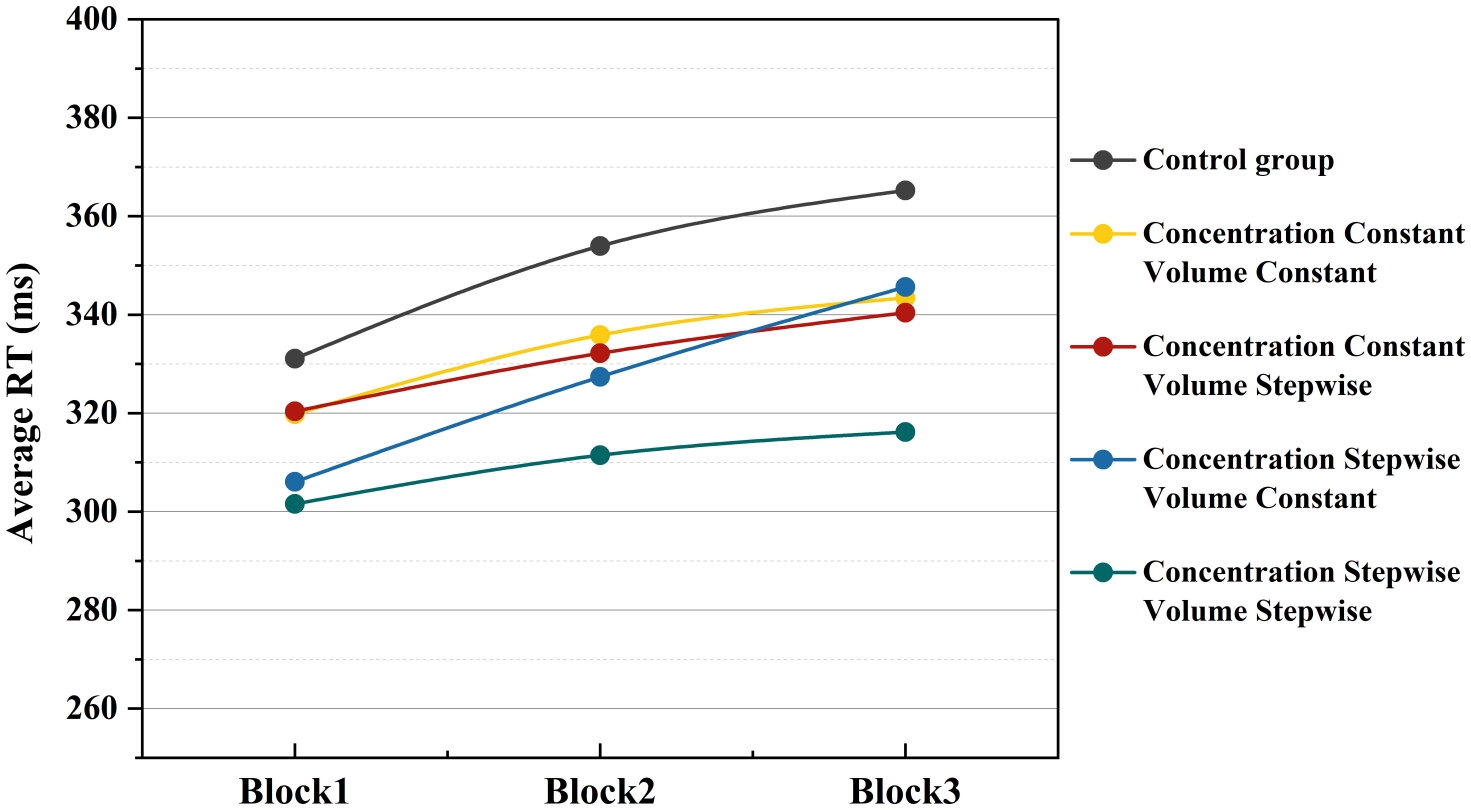
Temporal trend of mean RT under different experimental conditions.

To investigate the dynamic changes in behavioral alertness during the task process, this study further analyzed the temporal evolution of mean reaction time (RT) across three consecutive PVT task blocks (Block 1, Block 2, and Block 3). The trend of mean RT over time under each experimental condition is illustrated in Figure 6. In general, the mean RT under all experimental conditions showed varying degrees of increase as the task progressed. Among them, the control group exhibited the highest mean RT levels across all blocks, with the most pronounced upward trend. Compared to the control group, the RT curves for any condition containing music or odor stimulation were consistently lower overall, with a more gradual increase. Among the four intervention conditions, the experimental group with both incremental music and incremental odor maintained the lowest RT values throughout the task, showing the smallest increase across the three blocks, indicating the most stable alertness maintenance effect. The RT curves for the two single-increment conditions (music-increment only with constant odor and odor-increment only with constant music) fell between the dual-increment group and the dual-constant group: the differences from the dual-increment group were relatively small in the early task stage (Block 1), but as the task progressed, their RT values gradually increased, approaching or even exceeding those of the dual-constant group by Block 3. In contrast, the experimental group with both music and odor in constant mode (dual-constant condition) showed RT values that were already higher than other intervention groups at Block 1 and continued to rise in subsequent stages, exhibiting a trend close to that of the control group. This suggests that the constant stimulation mode has a relatively limited effect on maintaining alertness during prolonged tasks.

#### 4.3.3 Mean number of lapses

**Figure 7:**
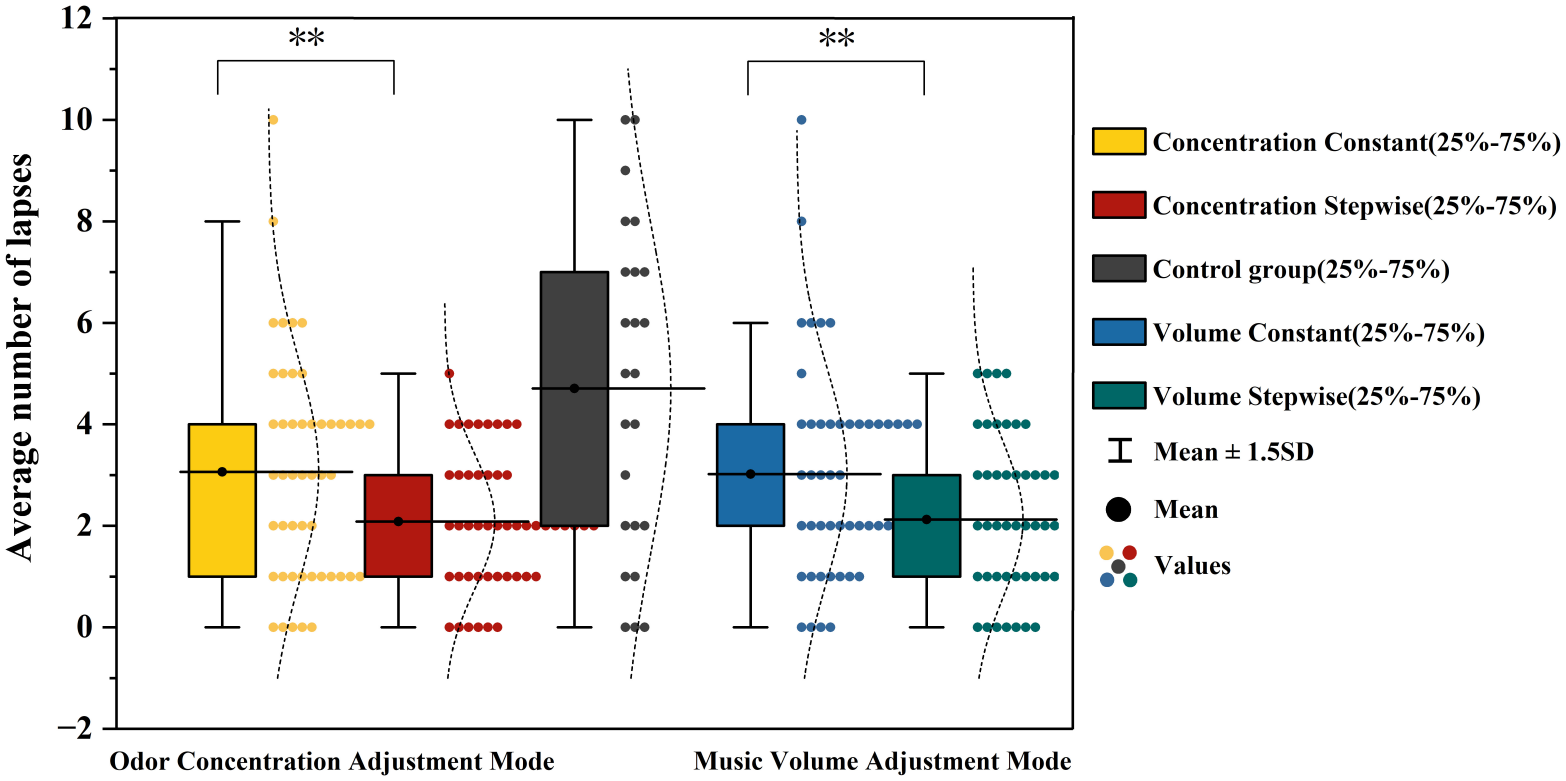
Analysis results of mean number of lapses under different music volume and odor concentration adjustment modes. **represents *p* < 0.01.

To further analyze the number of lapses in the Psychomotor Vigilance Task (PVT)—defined as trials with reaction times exceeding 500 ms or those with reaction times below 100 ms (typically considered invalid anticipatory responses)—as a supplementary behavioral metric, a repeated-measures analysis of variance (ANOVA) was conducted, with the no-music, no-odor blank control group serving as the baseline. The ANOVA results are presented in Table 3. The main effect of odor concentration adjustment mode was significant, *F*_(1, 23)_ = 9.668, *p* = 0.005, η*p*^2^ = 0.296. The mean number of lapses under the incremental concentration condition (M = 2.08, SD = 1.32) was lower than that under the constant concentration condition (M = 3.06, SD = 2.19). The main effect of music volume adjustment mode was also significant, *F*_(1, 23)_ = 8.774, *p* = 0.007, η*p*^2^ = 0.276, with fewer lapses in the incremental volume condition (M = 2.13, SD = 1.52) compared to the constant volume condition (M = 3.02, SD = 2.08). Pairwise comparisons revealed that, relative to the blank control group (M = 4.71, SD = 3.21), the number of lapses was lower under the music-incremental-only (*p* = 0.011), odor-incremental-only (*p* = 0.011), and dual-incremental (*p* = 0.002) conditions, but not under the dual-constant condition (*p* = 0.14). Among the four intervention conditions, the dual-incremental combination exhibited the lowest number of lapses. Further pairwise comparisons showed that the number of lapses in the dual-incremental condition was lower than in the dual-constant condition (*p* = 0.016). However, the differences between the dual-incremental condition and the music-incremental-only condition (*p* = 0.846), as well as the odor-incremental-only condition (*p* > 0.999), did not reach statistical significance. Specific comparisons of the mean number of lapses across different adjustment modes are illustrated in Figure 7.

**Table 3:** ANOVA results of mean number of lapses under music volume and odor concentration adjustment modes.

Factor	M	SD	95% CI	*F*	*df*1	*df*2	*p*	*ηp^2^*
**Control group**	4.71	3.21	(3.35, 6.06)					
**Odor concentration adjustment mode**				9.668	1	23	0.005^**^	0.296
Concentration constant	3.06	2.19	(2.42, 3.70)					
Concentration stepwise	2.08	1.32	(1.70, 2.47)					
**Music volume adjustment mode**				8.774	1	23	0.007^**^	0.276
Volume constant	3.02	2.08	(2.42, 3.62)					
Volume stepwise	2.13	1.52	(1.69, 2.57)					
Music volume adjustment mode ^*^ odor concentration adjustment mode				1.881	1	23	0.183	0.076

F: M, Mean; SD, Standard Deviation; 95% CI, 95% Confidence Interval; *F*-value; df, degrees of freedom; *p*, significance level; ηp^2^, partial eta-squared effect size; *p-*values are marked as follows:

^*^p < 0.05,

^**^p < 0.01,

^***^p < 0.001.

#### 4.3.4 Temporal changes in mean number of lapses

**Figure 8:**
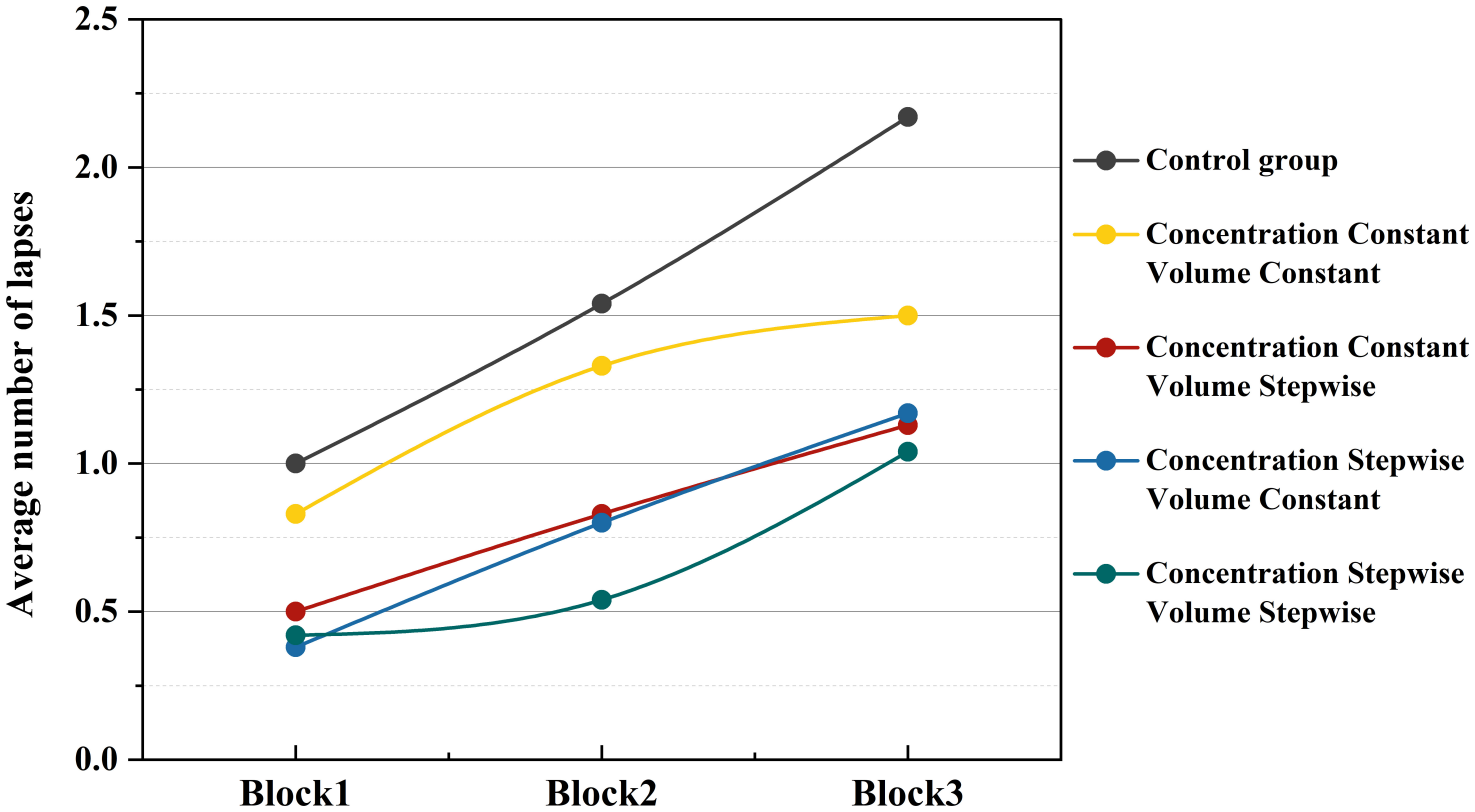
Temporal trend of mean number of lapses under different experimental conditions.

To further explore the dynamic changes in attentional lapses during the task, the temporal evolution of the mean number of lapses across the three consecutive PVT task blocks (Block 1, Block 2, and Block 3) was analyzed. As shown in Figure 8, the number of lapses under all experimental conditions exhibited an increasing trend as the task progressed, indicating a decline in attentional stability over time. The blank control group consistently showed the highest number of lapses at each stage, with the most rapid increase over time. In contrast, all conditions involving music or odor stimulation displayed a more gradual increase in lapse rate. Among the four intervention conditions, the dual-incremental group (in which both music volume and odor concentration were presented in incremental mode) maintained the lowest number of lapses throughout the task, reflecting the strongest maintenance of attentional stability. The single-incremental conditions (incremental music only or incremental odor only) showed lapse counts comparable to the dual-incremental group during the initial phase (Block 1), but these increased more noticeably as the task progressed. The dual-constant condition, in contrast, exhibited higher lapse counts from Block 1 onward, with a continued upward trend in subsequent blocks.

### 4.4 Physiological results (HF)

#### 4.4.1 Mean HF values

**Figure 9:**
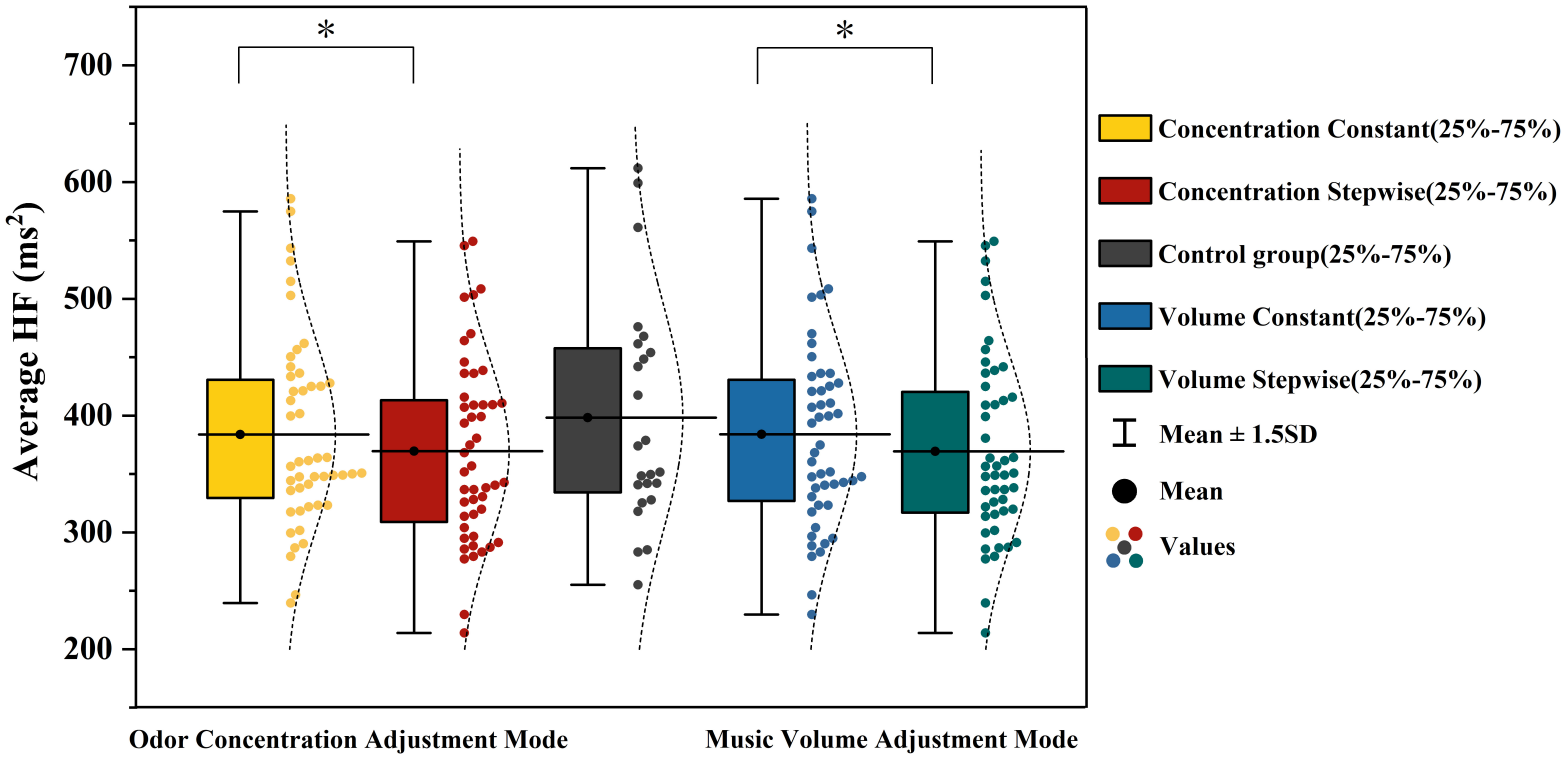
Analysis results of mean HF under different music volume and odor concentration adjustment modes. *represents *p* < 0.5.

Physiological alertness was assessed using high-frequency heart rate variability (HF). The results of the repeated-measures ANOVA are presented in Table 4. The control group (no music, no odor) exhibited the highest mean HF value (M = 398.28, SD = 96.94). A significant main effect was found for odor concentration adjustment mode, *F*_(1, 23)_ = 5.093, *p* = 0.034, η*p*^2^ = 0.181. HF values under the constant concentration condition (M = 383.76, SD = 81.40) were higher than those under the incremental concentration condition (M = 369.59, SD = 79.50). Similarly, the main effect of music volume adjustment mode was significant, *F*_(1, 23)_ = 4.412, *p* = 0.044, η*p*^2^=0.166, with higher HF values in the constant volume condition (M = 383.99, SD = 81.75) compared to the incremental volume condition (M = 369.36, SD = 79.11). Pairwise comparisons revealed that none of the experimental conditions—including the dual-constant, music-incremental-only, odor-incremental-only, and dual-incremental conditions—differed significantly from the control group in HF values (all *p* > 0.05). Furthermore, HF values under the dual-incremental condition did not significantly differ from those under the dual-constant condition, the music-incremental-only condition, or the odor-incremental-only condition (all *p* > 0.05). Specific comparisons of mean HF values across different adjustment modes are illustrated in Figure 9.

**Table 4:** ANOVA results of mean HF under music volume and odor concentration adjustment modes.

Factor	M	SD	95% CI	*F*	*df*1	*df*2	*p*	*ηp2*
**Control group**	398.28	96.94	(357.34, 439.21)					
**Odor concentration adjustment mode**				5.093	1	23	0.034^*^	0.181
Concentration constant	383.76	81.40	(360.12, 407.39)					
**Concentration stepwise**	369.59	79.50	(346.51, 392.68)					
Music volume adjustment mode				4.412	1	23	0.044^*^	0.166
Volume constant	383.99	81.75	(360.26, 407.73)					
Volume stepwise	369.36	79.11	(346.39, 392.33)					
Music volume adjustment mode ^*^ odor concentration adjustment mode				0.765	1	23	0.391	0.032

F: M: Mean; SD: Standard Deviation; 95% CI: 95% Confidence Interval; F-value; df: degrees of freedom; p: significance level; ηp^2^: partial eta-squared effect size; *p-*values are marked as follows:

^*^p < 0.05,

^**^p < 0.01, ^***^p < 0.001.

#### 4.4.2 Temporal changes in mean HF scores

**Figure 10:**
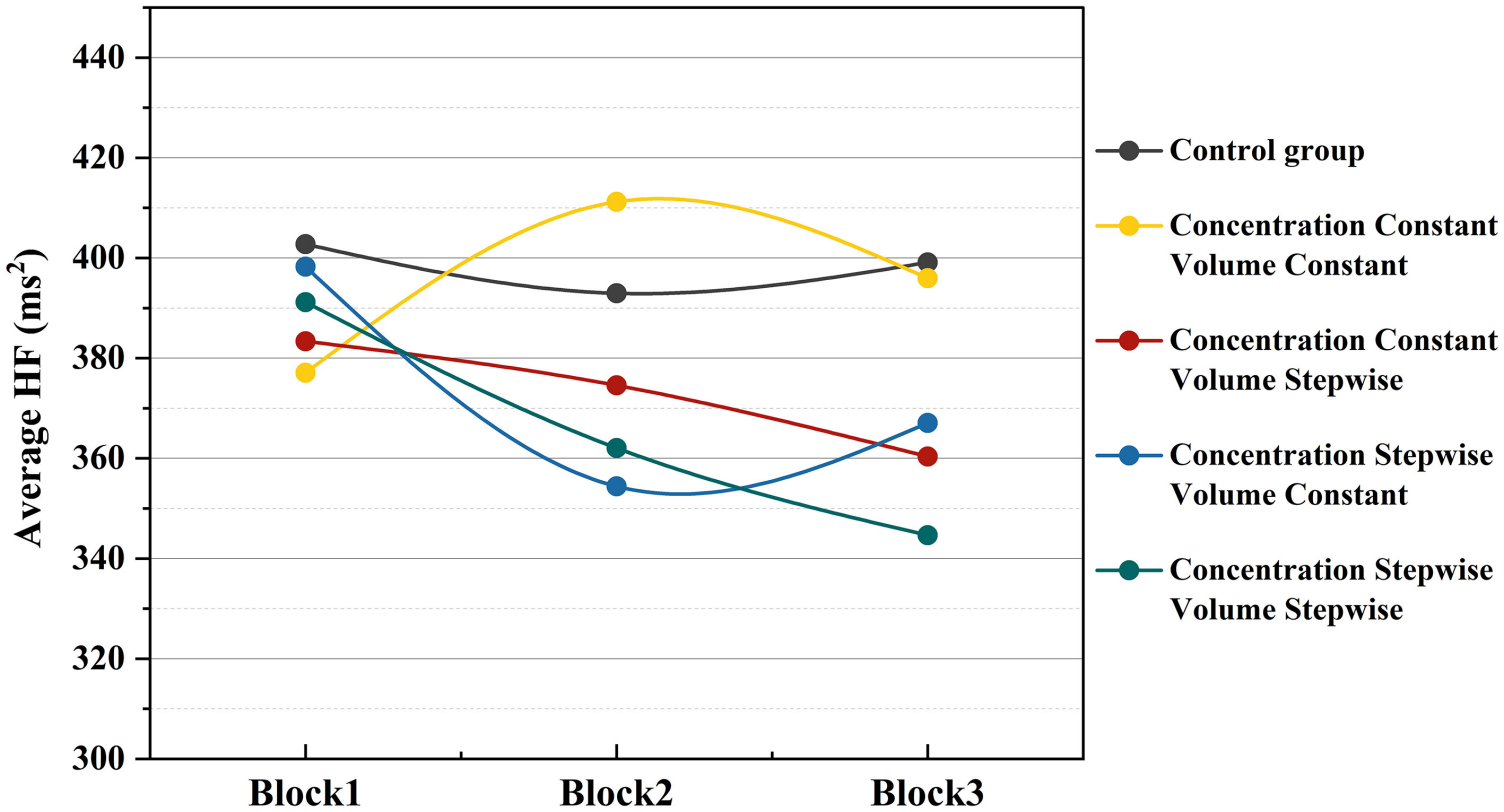
Temporal trend of mean HF under different experimental conditions.

To investigate the dynamic changes in physiological alertness during the experimental procedure, this study further analyzed the temporal trend of high-frequency heart rate variability (HF) across the task. As shown in Figure 10, the mean HF values under all experimental conditions exhibited distinct change patterns as the PVT progressed. The HF values for the blank control group remained at a relatively high level throughout the task, showing a fluctuating upward trend over time. In contrast, the HF curves for any experimental group that included either music or odor stimulation were consistently lower overall than that of the control group. Under the dual-incremental condition (both incremental music and incremental odor), HF values remained the lowest throughout the entire task, demonstrating the most rapid decreasing trend. The HF values for the single-incremental conditions (incremental music only or incremental odor only) fell between those of the dual-incremental group and the dual-constant group. The HF values under the dual-constant condition were slightly higher than those of the single-incremental groups and, during Block 2, even exceeded those of the blank control group, though they remained lower than the control group overall.

## 5 Discussion

This study systematically investigated the effects of music volume and peppermint odor concentration under constant vs. dynamic incremental adjustment modes on vigilance states by integrating subjective scales (KSS), behavioral performance measures (PVT reaction time and number of lapses), and physiological indicators (high-frequency heart rate variability, HF). Within the null-hypothesis significance testing framework, the reported *p-*values indicate the probability of observing the data (or more extreme data) under the assumption that the null hypothesis is true, not the probability that the null hypothesis itself is true (Gigerenzer, 2004). Therefore, our interpretations focus more on a comprehensive evaluation—integrating *p-*values, effect sizes (partial η^2^), the direction of mean differences, and the consistency of the observed patterns with our theoretical framework—rather than treating statistical significance alone as proof of the research hypotheses. The experimental results revealed that the dynamic incremental mode was consistently associated with advantages across subjective, behavioral, and physiological measures, supported by moderate-to-large effect sizes and converging patterns of results. Under incremental conditions, participants reported reduced subjective sleepiness, showed faster reaction times and fewer lapses, and displayed a statistically reliable decrease in HF, a pattern consistent with heightened physiological arousal. Notably, when both auditory and olfactory stimuli were presented incrementally, these improvements were generally more pronounced, with the dual-incremental condition numerically outperforming the single-incremental conditions on most metrics, although the statistical significance of this additive effect varied across indicators. These findings indicate that, compared to the blank control condition, any intervention involving auditory or olfactory stimulation was associated with improved vigilance outcomes. More importantly, for both modalities, the incremental presentation mode was more effective than the constant mode in sustaining vigilance over time. Furthermore, the dual-incremental condition yielded the most favorable overall vigilance state, a pattern consistent with an additive effect across sensory modalities, suggesting a potential additive benefit that warrants further investigation. This study not only provides empirical evidence consistent with the potential of sensory stimulation in modulating vigilance but also, for the first time within an auditory-olfactory integration framework, offers insights into the role of the dynamic incremental presentation mode in counteracting sensory adaptation and maintaining long-term vigilance.

(1) This study assessed the effects of music volume and odor concentration under constant and incremental adjustment modes on subjective vigilance using the Karolinska Sleepiness Scale (KSS). Repeated-measures ANOVA revealed significant main effects for both the music volume adjustment mode (*p* < 0.001, η*p*^2^ = 0.531) and the odor concentration adjustment mode (*p* < 0.001, η*p*^2^ = 0.452). KSS scores under the incremental mode were significantly lower than those under the constant mode. This pattern is consistent with the theoretical expectation formulated in H2a and H2b. Specifically, under the incremental music condition, the mean KSS score was 4.79, compared to 5.23 under the constant music condition. Similarly, under the incremental odor condition, the mean KSS score was 4.78, compared to 5.24 under the constant odor condition. These quantitative results indicate that the dynamic incremental presentation mode is more effective in reducing subjective sleepiness and enhancing alertness. The core mechanism underlying this finding lies in counteracting sensory adaptation (Mackworth, 1968; Jefferis et al., 2024). During sustained tasks, constant auditory or olfactory input leads to a gradual reduction in the responsiveness of sensory receptors and central neural pathways, i.e., habituation, which weakens their ability to maintain arousal and attention (Mackworth, 1968). This notion of rapid neural habituation in high-order cortical areas is supported by converging evidence. For instance, in the visual domain, Gaillard et al. (2006) demonstrated using both fMRI and direct intracranial recordings that the left occipitotemporal cortex—a region critical for visual word form recognition—exhibits a rapid and selective adaptation to repeated alphabetic stimuli. This finding underscores the general principle that cortical sensory systems quickly reduce their response to constant or repeated input, a mechanism that likely underlies the diminished efficacy of fixed-intensity stimuli in sustaining vigilance over time. Moreover, from the perspective of arousal theory, an individual's vigilance level exhibits a classic “inverted-U-shaped” relationship with the intensity of external stimuli (Yerkes and Dodson, 1908). Constant stimulation, even if initially set at an optimal intensity, tends to gradually deviate from this optimal zone due to sensory adaptation, leading to under-arousal. In contrast, the dynamic incremental mode, by phasically increasing stimulus intensity, not only disrupts the adaptation cycle but also helps to dynamically maintain an individual's perceptual and physiological arousal levels within the peak region of the inverted-U curve. This likely results in an optimized subjective arousal experience (Yerkes and Dodson, 1908; Esterman and Rothlein, 2019), as the stimulation intensity is maintained within a more appropriate range throughout the task. The incremental mode adopted in this study, by progressively increasing stimulus intensity, effectively disrupts this adaptive cycle, thereby delaying the decline in vigilance. This aligns with observations by Li et al. (2025b) in dynamic lighting and Ma et al. (2026) in dynamic odor concentration studies, where dynamic stimuli showed superior advantages in maintaining long-term performance. Notably, when both music and odor were presented in the incremental mode, the lowest KSS scores were observed across all experimental conditions. The dual-incremental condition consistently outperformed all other conditions. Specifically, KSS scores under this condition were significantly lower than those of the blank control and dual-constant conditions and were numerically lower than those of either single-modality incremental condition. This pattern suggests that the benefits of incremental auditory and olfactory stimulation may combine additively rather than interactively. The underlying mechanism can be understood from two complementary perspectives. First, within each sensory modality, the incremental mode delays sensory adaptation by phasically increasing stimulus intensity, thereby maintaining neural responsiveness over time (Mackworth, 1968; Jefferis et al., 2024). Second, when both modalities are presented in incremental mode, these modality-specific benefits likely accumulate, resulting in enhanced overall subjective alertness (Li et al., 2025a). This additive effect may reflect independent contributions from auditory and olfactory pathways to common arousal networks, such as the locus coeruleus-norepinephrine system (Sara, 2009). These findings indicate that combining dynamic stimuli across modalities can yield cumulative benefits for vigilance maintenance.
(2) This study examined the effects of music volume and odor concentration under constant vs. incremental adjustment modes on behavioral vigilance, as measured by reaction time (RT) and the number of lapses in the Psychomotor Vigilance Task (PVT). Repeated-measures ANOVA revealed a significant main effect of odor concentration adjustment mode on mean RT (*p* = 0.002, η*p*^2^ = 0.338), and a significant main effect of music volume adjustment mode (*p* = 0.007, η*p*^2^ = 0.273). Mean RT under the incremental concentration condition (321.43 ms) was significantly shorter than under the constant concentration condition (334.70 ms); similarly, mean RT under the incremental volume condition (322.72 ms) was significantly shorter than under the constant volume condition (333.41 ms). For the number of lapses, the main effect of odor concentration adjustment mode was significant (*p* = 0.005, η*p*^2^ = 0.296), with fewer lapses in the incremental concentration condition (2.08) compared to the constant concentration condition (3.06). The main effect of music volume adjustment mode was also significant (*p* = 0.007, η*p*^2^ = 0.276), with fewer lapses in the incremental volume condition (2.13) than in the constant volume condition (3.02). These findings indicate that, for both auditory and olfactory modalities, the incremental adjustment mode was associated with better behavioral performance in terms of faster response speed and greater response stability. This pattern of results aligns with the predictions of H2a and H2b. From a cognitive and neural mechanism perspective, constant stimulation tends to induce vigilance decrement, which is commonly attributed to sensory adaptation and the gradual depletion of central attentional resources (Mackworth, 1968; Dinges and Powell, 1985). In this study, the incremental mode, by enhancing stimulus intensity in stages, may have re-engaged attentional resources and delayed neural habituation, which could account for the observed superior behavioral performance. Additionally, this strategy not only counters sensory adaptation but also aligns with the “inverted-U-shaped” relationship described by arousal theory. Specifically, through dynamic adjustment, the stimulus intensity is continually maintained around the optimal arousal zone, thereby preventing prolonged fixed-intensity stimulation from causing arousal levels to drift toward either end of the curve (under- or over-arousal). As a result, vigilance can be sustained more stably at the behavioral level (Yerkes and Dodson, 1908; Esterman and Rothlein, 2019). This finding provides empirical evidence that is consistent with the theoretical framework proposing that dynamic stimuli can counteract sensory adaptation and sustain neural arousal. The behavioral advantage of the incremental mode can be explained by the mechanism of resisting sensory adaptation: constant stimulation readily leads to a decline in perceptual system responsiveness (Mackworth, 1968), whereas phased intensification of stimuli may reactivate attention and delay this process. The incremental volume levels employed in the auditory channel (50–70 dB) directly address previous discussions on appropriate volume ranges, avoiding both under-arousal at low volumes and potential performance impairment at excessively high volumes (Dalton et al., 2008; Turner et al., 1996), while dynamically extending the vigilance-promoting effect maintained by moderate volume (Mizoguchi and Tsugawa, 2012). However, it is important to acknowledge that music is not merely an acoustic signal but a complex stimulus embedded with cultural and semantic meaning (Sun et al., 2024). Although steps were taken to minimize the influence of lyrical content—such as familiarizing participants with the music during practice and instructing them to treat it as background—the potential confounding effects of the song‘s genre and narrative cannot be entirely ruled out (Sun et al., 2024). It is possible that stylistic familiarity or emotional resonance subtly modulated participants' engagement. Given that the incremental mode varied only intensity while keeping musical content constant, future studies should test whether these effects generalize across different genres or can be replicated with semantically neutral stimuli (e.g., pure tones) to disentangle acoustic dynamics from higher-order cognitive processing. Similarly, the incremental concentration pattern in the olfactory channel (1%−30%) echoes the concentration-dependent regulation of vigilance (Shi et al., 2025) and, through phased enhancement, circumvents the gradual decline in efficacy due to olfactory adaptation that may occur with fixed concentrations (Tang, 2022). Moreover, from the perspective of attention resource models (Wahn and König, 2016), constant stimulation may lead to rapid depletion of modality-specific attentional capacity due to repetitive engagement of the same neural pathways. In contrast, dynamic multimodal stimulation can redistribute load across auditory and olfactory channels, thereby reducing the risk of overloading a single modality and preserving overall attentional resources. This resource-based interpretation complements the sensory adaptation account and further supports the behavioral benefits observed under incremental conditions. Thus, the incremental mode is not merely an additive increase in intensity but operates through dynamic adjustment to counteract adaptation, thereby more stably sustaining vigilance at the behavioral level, offering a potential mechanistic explanation for the behavioral advantages observed under H2a and H2b. Consistent with H1, all experimental conditions involving music or odor stimulation significantly shortened RT compared to the blank control condition (all *p* < 0.05). For lapses, the music-incremental-only, odor-incremental-only, and dual-incremental conditions also showed significantly fewer lapses (all *p* < 0.05), whereas the reduction under the dual-constant condition did not reach statistical significance (*p* = 0.14). Overall, these findings suggest that auditory and olfactory stimuli can contribute to enhanced behavioral vigilance, particularly under incremental presentation modes. More importantly, in support of H3, the dual-incremental condition (incremental music + incremental odor) produced the shortest RT and the fewest lapses among all conditions. Pairwise comparisons showed that the dual-incremental condition significantly outperformed the dual-constant condition in both RT (*p* = 0.006) and lapse count (*p* = 0.016). Although the differences between the dual-incremental and single-incremental conditions (music-incremental-only or odor-incremental-only) did not reach statistical significance in lapse count (*p* > 0.05), the numerical trends consistently favored the dual-incremental condition across all behavioral metrics. This pattern suggests an additive enhancement effect when both modalities are presented incrementally, supporting the additive benefit of cross-modal dynamic stimulation in sustaining behavioral vigilance. For the number of lapses, although the dual-incremental condition showed numerically lower values than the single-increment conditions, the difference did not reach statistical significance. This may be partly attributable to the relatively small sample size of this study, which limited the statistical power to detect subtle differences. As a low-frequency event, the variance in lapse counts is more susceptible to individual differences and random factors, which may weaken the sensitivity of statistical tests with a limited sample (Costello and Watts, 2017; Duffy and Smith, 2018). On the other hand, this may also imply that the facilitative effect of cross-modal dynamic combination on behavioral performance is primarily reflected in accelerated information processing speed (i.e., shortened reaction time), whereas its enhancement of attentional control functions that suppress occasional errors (i.e., reducing lapses) may indeed be limited, or may require greater differences in stimulus intensity for the additive effect to become apparent (Chan and Dyson, 2015). On the other hand, it may also suggest that the promoting effect of bimodal dynamic synergy on behavioral performance is primarily manifested in the acceleration of information processing speed, while its enhancement of attentional control functions that suppress occasional errors may be more limited, or may require greater differences in stimulus intensity to become apparent.
(3) This study further explored the effects of different sensory stimulation modes on physiological alertness from the perspective of autonomic nervous system regulation. High-frequency heart rate variability (HF) serves as a reliable physiological indicator reflecting the strength of parasympathetic nervous activity. A decrease in HF is typically associated with increased attentional focus, elevated cognitive load, and enhanced alertness (Zhou and Zhou, 2022; Kang et al., 2024). The experimental results demonstrated that both the music volume adjustment mode and the odor concentration adjustment mode had a significant impact on HF values. Specifically, HF values under the incremental mode were significantly lower than those under the constant mode (*p* < 0.05), providing physiological support for hypotheses H2a and H2b. In concrete terms, under the incremental music volume condition, the mean HF value was 369.36 ms^2^, significantly lower than the 383.99 ms^2^ under the constant volume condition. Similarly, under the incremental odor concentration condition, the mean HF was 369.59 ms^2^, also significantly lower than the 383.76 ms^2^ under the constant concentration condition. This physiological trend showed high synchrony with changes in subjective alertness (decreased KSS scores) and behavioral performance (shortened RT and reduced lapses). This consistency across multimodal measures not only supports the role of the incremental mode in delaying sensory adaptation, but also indicates, from the perspective of arousal regulation, that dynamically increasing stimuli help maintain autonomic nervous activity in a more optimal state of arousal. That is, it helps avoid excessive parasympathetic activation (under-arousal) or sympathetic overactivation (over-arousal), thereby optimizing the physiological level of vigilance associated with task performance (Yerkes and Dodson, 1908; Esterman and Rothlein, 2019). This indicates that the incremental stimulation mode not only enhanced individuals' subjective alertness and behavioral performance but also induced significant changes in autonomic nervous system regulation at the physiological level. From a neurophysiological mechanism perspective, the decrease in HF values likely reflects the efficacy of the incremental mode in countering sensory adaptation and maintaining central nervous system arousal. During prolonged tasks, constant stimuli tend to induce a state of parasympathetic dominance, manifested as increased HF values, which is subsequently accompanied by attentional dispersion and declining alertness (Zhou and Zhou, 2022; Kang et al., 2024). In contrast, the incremental increase in music volume and odor concentration, by phasically enhancing sensory input, may potentiate sympathetic modulation, thereby inhibiting parasympathetic activity and resulting in a significant decrease in HF. This finding aligns with the conclusions of Zhou et al. (Zhou and Zhou, 2022) on the association between HRV and alertness states, namely that lower HF values correlate with higher alertness and greater cognitive load. It is particularly noteworthy that under the condition where both odor concentration and music volume were presented in the incremental mode (dual-incremental), the differences did not reach statistical significance. This suggests that when both auditory and olfactory modalities employ a dynamic incremental mode, a cross-modal additive inhibition may occur, further augmenting the activation of the sympathetic nervous system and thereby demonstrating a stronger alertness-sustaining effect in the physiological measures. This finding is consistent with the possibility of an additive effect as proposed in H3, although further studies are required to examine this mechanism. From a neuroergonomics perspective (Mehta and Parasuraman, 2013), this reduction in HF may also reflect engagement of central arousal systems that could be periodically reactivated by dynamic sensory input to counteract neural fatigue. Such findings support the potential for integrating real-time neurophysiological monitoring with adaptive multimodal interventions in safety-critical environments. Although the difference in HF values between the dual-incremental condition and the single-incremental conditions was not statistically significant, this may be related to the relatively small sample size and the considerable inter-individual variability in autonomic nervous system responses. Furthermore, as an indicator of parasympathetic activity, variations in HF can also be modulated by other factors such as breathing patterns and emotional state (Bernardi et al., 2000). An observation worth noting is that under the dual-constant condition, HF values exhibited an anomalous increase during Block 2, even exceeding those of the blank control group. This pattern may reflect pronounced sensory adaptation to unchanging stimuli, leading to a transient rebound in parasympathetic activity or even temporary attentional disengagement from the task. Alternatively, this physiological “paradoxical rise” could be influenced by unmonitored variations in respiratory patterns, highlighting the need for stricter respiratory control in future studies to isolate HF changes attributable to autonomic arousal (Bernardi et al., 2000).

This study systematically examined the effects of music volume and odor concentration under constant vs. incremental modes on vigilance states by integrating subjective ratings (KSS), behavioral performance (PVT reaction time and number of lapses), and physiological indicators (HF-HRV). Compared to the control condition, the data show higher vigilance levels when auditory or olfactory stimulation is present. This observation aligns with the theoretical expectation described in H1. Across subjective, behavioral, and most physiological measures, the incremental mode showed stronger effects than the constant mode. This pattern is compatible with the theoretical expectations described in H2a and H2b, and most physiological measures, with significant main effects and moderate-to-large effect sizes. Regarding H3, a pattern of additive benefit was observed when both modalities were presented incrementally: the dual-incremental condition yielded the best overall vigilance performance, particularly evident in reaction time and subjective alertness, and significantly outperformed the dual-constant condition on most metrics. However, its advantage over single-incremental conditions did not consistently reach statistical significance across all measures, suggesting that while cross-modal dynamic stimulation shows promise for enhancing vigilance, the additive effects may be metric-dependent and warrant further investigation. These findings confirm that the dynamic incremental mode can effectively counteract vigilance decline during prolonged tasks by delaying sensory adaptation and sustaining neural arousal within a multisensory integration framework, providing both theoretical and empirical foundations for optimizing vigilance intervention strategies.

## 6 Conclusion

This study systematically examined the effects of music volume and odor concentration on vigilance under constant and incremental adjustment modes, using subjective ratings, behavioral performance, and physiological measures. Grounded in sensory adaptation and arousal theories, the findings highlight the advantage of a dynamic incremental stimulation mode in sustaining long-term vigilance. Compared to a no-stimulation baseline, all interventions involving music or odor enhanced subjective alertness, improved reaction time and response stability in the Psychomotor Vigilance Task (PVT), and were associated with reduced high-frequency heart rate variability (HF), indicating increased physiological arousal. Further analysis showed that, for both auditory and olfactory modalities, the incremental mode was more effective than the constant mode in maintaining vigilance over time, primarily by delaying sensory adaptation and sustaining central nervous system arousal. Notably, when music and odor were presented together in an incremental fashion, a cross-modal additive effect emerged: participants showed the highest levels of vigilance across multiple indicators. These results suggest that dynamic multisensory integration may offer a promising approach to counteracting vigilance decrement during prolonged tasks.

Despite these findings, this study has several limitations. While measures were taken to minimize semantic influences, the potential confounding effects of the song's lyrics and cultural context cannot be entirely ruled out. Moreover, using a single musical stimulus limits generalizability across auditory environments and genres. The sample consisted primarily of young, healthy adults; future research should include more diverse age groups, genders, and occupational backgrounds. The 10-minute PVT, while sensitive to early vigilance decline, is relatively short; longer tasks could better capture vigilance trajectories over time. The controlled laboratory environment, though ensuring internal validity, may limit applicability to real-world settings involving distractions and multitasking. Additionally, the display was not photometrically calibrated, so specific luminance values are unavailable, though brightness and contrast were held constant across sessions to maintain consistency. Future studies should calibrate displays to enable precise stimulus control. Respiratory rate was not objectively monitored, which may have introduced variability in HF-HRV; stricter respiratory control is recommended. The 1-min pre-task odor release, intended to establish a baseline, may have inadvertently reduced odor intensity during the initial task block, potentially underestimating the incremental mode's benefits. Ideally, a calibrated olfactometer should be used for precise odor delivery. The incremental mode was confounded with time on task, as intensity increased with successive PVT blocks; future designs should incorporate randomized or decreasing-intensity conditions to isolate dynamic effects. The absence of direct neural measures (e.g., EEG, fNIRS) limits mechanistic interpretation of cortical adaptation or LC-NE involvement. Future work should explore diverse music genres, odor types, and parameter combinations, as well as individual differences such as personality, baseline vigilance, and sensory sensitivity, which may moderate intervention effects. Although the present short-term PVT results provide preliminary evidence for the efficacy of dynamic increments, further studies are needed to validate these effects in ecologically valid settings and with longer tasks. By demonstrating that dynamic, incremental increases in sensory intensity can effectively counteract vigilance decrement within a controlled laboratory setting, this study contributes to a foundational understanding of the relationship between sustained attention and environmental stimulation. Rather than viewing sensory input as a static resource, our findings, specific to these experimental conditions, support a model in which adaptive modulation of the sensory environment can influence cognitive and physiological states. This work provides an empirical basis for future research aimed at developing more sophisticated, context-aware interventions, although substantial investigation remains necessary before such strategies can be considered for application in safety-critical domains.

## Data Availability

The datasets presented in this study can be found in online repositories. The names of the repository/repositories and accession number(s) can be found below: https://doi.org/10.57760/sciencedb.37092, Science Data Bank.
